# Copper(II) complexes as potential anticancer and Nonsteroidal anti-inflammatory agents: *In vitro and in vivo studies*

**DOI:** 10.1038/s41598-019-41063-x

**Published:** 2019-03-27

**Authors:** Afzal Hussain, Mohamed Fahad AlAjmi, Md. Tabish Rehman, Samira Amir, Fohad Mabood Husain, Ali Alsalme, Maqsood Ahmad Siddiqui, Abdulaziz A. AlKhedhairy, Rais Ahmad Khan

**Affiliations:** 1 Department of Pharmacognosy, College of Pharmacy, King Saud University, P.O. Box 2457, Riyadh, 11451 Saudi Arabia; 2 Department of Chemistry, College of Science and General Studies, Alfaisal University, Riyadh, Saudi Arabia; 3 Department of Food Science and Nutrition, Faculty of Food and Agricultural Sciences, King Saud University, 2460, Riyadh, 11451 Saudi Arabia; 4 Department of Chemistry, College of Science, King Saud University, P.O. Box 2455, Riyadh, 11451 Saudi Arabia; 5 Al-Jeraisy Chair for DNA Research, Zoology Department, College of Science, King Saud University, Riyadh, 11451 Saudi Arabia

## Abstract

Copper-based compounds are promising entities for target-specific next-generation anticancer and NSAIDS therapeutics. *In lieu* of this, benzimidazole scaffold plays an important role, because of their wide variety of potential functionalizations and coordination modes. Herein, we report three copper complexes **1**–**3** with benzimidazole-derived scaffolds, a biocompatible molecule, and secondary ligands viz, 1–10-phenanthroline and 2,2′-bipyridyl. All the copper complexes have been designed, synthesized and adequately characterized using various spectroscopic techniques. *In-vitro*, human serum albumin (HSA) binding was also carried out using fluorescence technique and *in-silico* molecular modeling studies, which exhibited significant binding affinities of the complexes with HSA. Furthermore, copper complexes **1**–**3** were tested for biological studies, i.e., anticancer as well as NSAIDS. *In vitro* cytotoxicity results were carried out on cultured MCF-7 cell lines. To get the insight over the mechanism of action, GSH depletion and change in lipid peroxidation were tested and thus confirmed the role of ROS generation, responsible for the cytotoxicity of the complexes **1**–**3**. Moreover, the copper complexes **1**–**3** were tested for potential to act as NSAIDS on albino rats and mice in animal studies *in-vivo*. Additionally, we also predicted the mechanism of action of the copper complexes **1**–**3** using molecular modeling studies with COX-2 inhibitor.

## Introduction

The essential trace element-copper has a key role in numerous physiological cellular processes. However, its high redox activity makes the free copper ions highly cytotoxic, and thus the intracellular level of copper must need to be strongly regulated^1^. It is well known that in neoplastic diseases, copper metabolism is severely altered. The connection between copper elevated level in serum progression, tumor burden and recurrence in a variety of cancers viz, liver, lung, prostrate, breast, sarcoma, Hodgkin’s lymphoma is well documented^2–4^. However, the molecular mechanism between the elevation in copper concentration and malignant cells is still not known exactly. It can only be hypothesized particularly at early stages after considering the role copper plays in tumor angiogenesis in early stages^5^. In angiogenesis, copper seems to be involved in stimulating proliferation of endothelial cells migration and acts as a cofactor for VEGF, bFGF, TNF-α, and IL1 (Angiogenic factors)^5–8^. Also, in embryogenic cells, human copper transporter (hCTR1) curbs cell signaling pathways activation, thus leads to the development and progression of cancers^9^. The difference in response of tumor cell and normal cell towards copper possibly laid the foundation of copper complexes evolution as anticancer agents. However, a large number of copper complexes with various sets of ligands have been prepared for this cause and displayed noteworthy *In vitro* cytotoxicities^10–16^. But, very few copper complexes have been tested for preclinical *in vivo* modules^14–16^.

Pain is a defensive alarm in our body to notify us of any injuries or diseases. The chronic pain significantly alters the quality of life of individuals, and nearly every individual suffers from pain varying from acute to chronic, worldwide^17,18^. Thus, it is a major challenge to treat pain, in particular, chronic pain^19^. Therefore, there is always a necessity to develop novel analgesic/anti-inflammatory drugs with improved efficacy and lesser side effects. Numerous NSAIDs (i.e., non-steroidal anti-inflammatory drugs) have been used more often to cure numerous chronic inflammatory diseases^20^. In general, the mode of action of NSAIDs is by restricting prostaglandin biosynthesis whereas as in case of some pro-inflammatory, inhibition of cyclooxygenase (COX) enzyme^21^, and thus accountable of transforming arachidonic acid into prostaglandins^22,23^. The use of metal ions along with NSAIDs such as copper ibuprofenate (a compound of copper and ibuprofen chelating agent) has been reported better drugs then the parent compounds^24,25^. It has shown that copper ibuprofenate complex exhibited a more avid effect as compared to the parent drug^26^, with lesser gastrointestinal side-effects^27^. Several groups are working over the new NSAIDs designing and derivatization of the marketed NSAIDs to modify and/or improve the effectiveness of the drugs/potential drug candidates towards a selectively preferred function^28,29^.

For the development of metal complexes particularly copper, to be able to possess anticancer and NSAIDs activity, a crucial role is played by the organic motifs, its framework, and the donor atom set. The solubility and ability to cross cell membranes of the drug candidate are governed by the hard/soft nature of metal ion and the lipophilic/hydrophilic balance. The stability of the compound/potential drug candidate towards biomolecules is determined by the ligand(s). Thus, we have taken benzimidazole Schiff’s base as a primary ligand. The rigid guanidine pharmacophore of 2-aminobenzimidazole is an immunomodulator and in immune cells known to change the inducible form of Nitric Oxide (NO) synthetase. The guanidine motifs also are known to stimulate blood vessels dilation and leucocytes activation needed for action against tumor cells, fungi as well as bacteria. The benzimidazole’s derivative is known to have various properties viz, anti-viral, anti-proliferative, anti-infective, etc^30–33^. Benzimidazole motifs also inhibit Chemokine receptor (CXCR3), enzymes viz, ITK, interleukin 2 inducible T cell kinase and Lck, lymphocyte tyrosine kinase and thus immune system get altered^30^, *reftherein*. Also, benzimidazole motifs can easily undergo derivatization and can be functionalized accordingly and comprise of stable and biologically advantageous candidates.

Cancer causes chronic pain in the last stages, so our aims in this study to develop bifunctional molecules that can cure cancer simultaneously release the pain also. In this manuscript, we have synthesized an organic motif, Schiff base of 2-aminobenzimidazole with o-vanillin^34^ and three copper complexes using copper (II), Schiff base as a primary ligand (**1**), and with 1,10-phenanthroline and 2,2′-bipyridyl as co-ligand (**2** and **3**). Then, we have studied their potential to act as an anti-cancer chemotherapeutic as well as non-steroidal inflammatory drugs (NSAIDs) candidates using *In vitro* and *in vivo* studies.

## Results and Discussion

### Synthesis and Characterization

**Figure 1:**
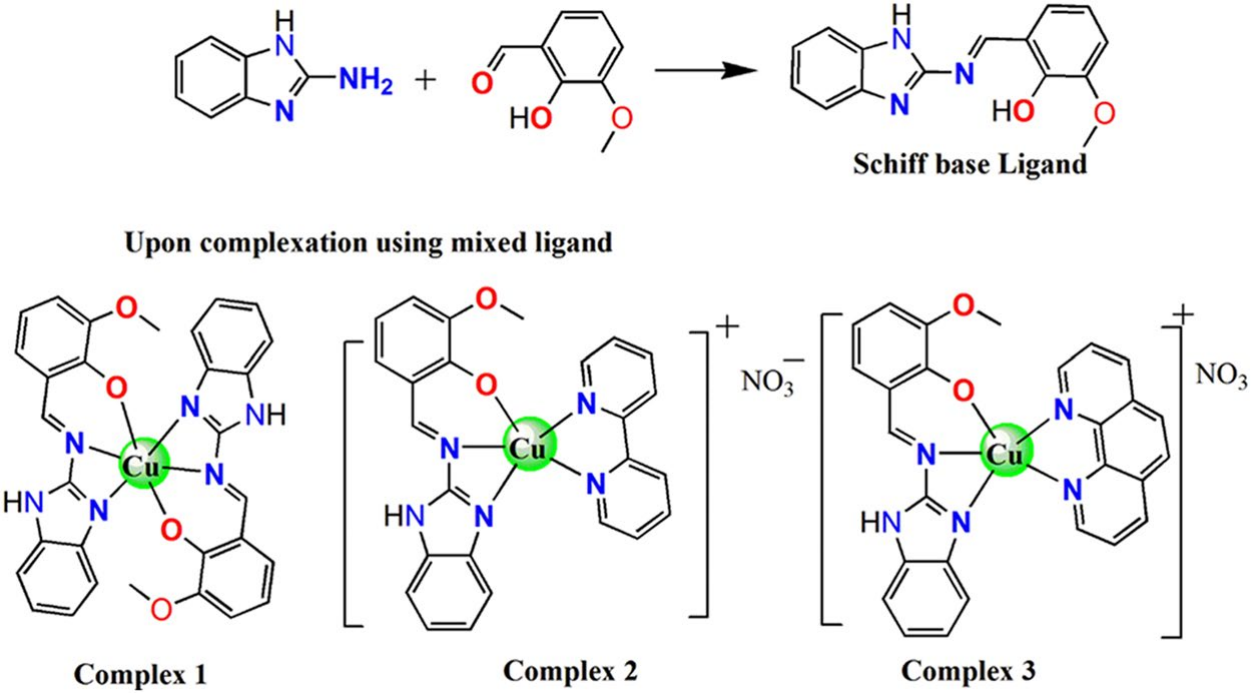
Outline of the synthesis of the Schiff base ligand and structures of the ternary complexes.

Three copper(II) complexes **1**–**3** are prepared in high yield from reactions of tridentate Schiff base–ONN- ligand (**L**) derived from 2-aminobenzimidazole and o-vanillin in the presence of few drops of acetic acid as reported earlier by our group^34^ with copper(II) salt like [Cu(NO_3_)_2_]∙3H_2_O and N,N-donor heterocyclic bases, viz. Bpy, (**2**) and Phen (**3**), as coligand in 1:1:1 ratio. These complexes were formulated as [Cu(L)_2_] (**1**), [Cu(L)(bpy)(H_2_O)] NO_3_ and [Cu(L)(Phen)(H_2_O)] NO_3_ (**2** and **3**), (Fig. 1).

The copper (II) complexes **1**–**3** are characterized by elemental analytical and various other spectroscopic techniques. The one-electron paramagnetic (µ_eff_ ~ 1.8 µ_B_) complexes show a d–d band in the electronic spectral range of 590–650 nm in 9:1 (v/v) H_2_O–DMSO mixture. The intense electronic bands observed near 300 nm are assignable to the π–π* intraligand transitions. The conductivity measurements indicate 1:1 electrolytic nature of the complexes **2** and **3** whereas **1** as non-electrolyte. These complexes are quite stable in solution even after 48 h, as confirmed by UV-Vis spectral studies.

The IR spectrum “ligand” L, exhibited characteristic band for azomethine C=N moiety at 1600 cm^−1^. Upon complexation, the IR spectra of the complexes **1**–**3** showed characteristic bands for the C=N moiety at ~1697–1690 cm^−1^^35^. In the spectra of the complexes, the ν(C=N) group of the ligand shifts of ~90 cm^−1^ from ~1600 cm^−1^ to ~1690 cm^−1^ indicating the coordination via the Schiff base azomethine nitrogen. Furthermore, the peaks around ~1610, 1519 cm^−1^ in complexes **2** and **3** are associated with ν(C=N) and ν(ArC=C) bands of the heterocyclic co-ligands viz, phen, bpy^36^. An intense band assigned to ν(NO_3_) is also evident at around 1383 cm^−1^, thus confirming the presence of the nitrate group^37^. The peak at 516 and 463 cm^−1^ in the ligand infrared spectrum is shifted to 518, 449 cm^−1^ (for **1**), 503, 428 cm^−1^ (for **2**) and 505, 427 cm^−1^ (for **3**), ascertained the coordination with Cu-O and Cu-N, respectively^38,39^ (See ESI Figs S1–S4).

The mass spectrum of **1** showed characteristic molecular ion peak at m/z 597 [for CuL_2_] ++ 2H^+^. Similarly, the mass spectra of complexes **2** and **3** showed characteristic ion peak at m/z values are 486 [M]^+^ + H^+^ and 510 [Cu + L + Phen]^+^  + 2H^+^, respectively. The mass spectra of the complexes **1**–**3** showed the parent ion peaks in aqueous DMSO suggesting the stability of the mononuclear ternary copper(II) species in the aqueous phase.

The EPR spectra of all the three copper(II) compounds in the solid state were recorded (spectra are depicted in ESI, Fig. S5). The observed g values lie in the expected range of Cu(II) complexes^40^, having g_av_ values of 2.089, 2.042 and 2.055 for compounds **1**, **2** and **3**, respectively. The phenomenon of hyperfine splitting and g-anisotropy value were not resolved, because of the relatively close proximity of the Cu(II) ions in the solid state. The magnetic moments obtained is in the range of 1.79–1.88 BM, which is consistent with the Cu^2+^ oxidation state with spin = ½.

### Biological Assays

The protein binding, molecular modeling, *In vitro* cytotoxic and *in vivo* analgesic, antipyretic and anti-inflammatory activity of the copper(II) complexes **1**–**3**, were studied to have a first insight on their potential anti-cancer and NSAIDs properties.

### *In vitro* Protein binding affinity of Copper complexes

#### Fluorescence quenching

**Figure 2:**
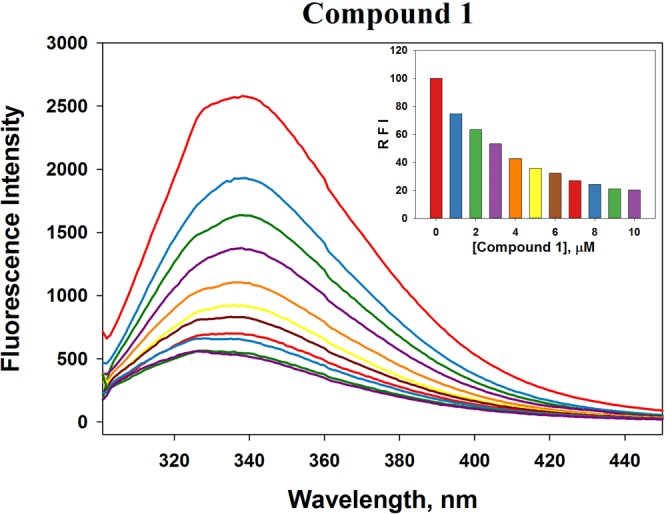
Quenching in the fluorescence of HSA in the presence of copper complex **1**. The inset shows a progressive decrease in the fluorescence intensity with increasing concentration of **1**.

HSA is a protein of choice by many biochemists to study drug-protein interaction and its contribution to the distribution and metabolism of the drug through different organs of the human body^41^. The strong fluorescence property of HSA is attributed to the presence of the only Trp-214 residue at Sudlow’s site II (sub-domain IIA). In this study, we have monitored the quenching in the fluorescence properties of HSA in the presence of metal complexes (Figs 2 and S6). It is clear that the fluorescence intensity of HSA was decreased progressively with the binding of copper complex 1 (Fig. 2 inset). The fluorescence intensity of HSA at 338 nm (λ_max_) was decreased significantly to 30% when the molar ratio of HSA: metal complex was 1:5. The quenching parameters were deduced using the following Stern-Volmer (Eq. 1) and modified Stern-Volmer (Eq. 2) equations:

1 $$\frac{{F}_{o}}{F}=1+{K}_{SV}[Q]=1+{k}_{q}{\tau }_{o}[Q]$$

where, *Fo* and *F* are the fluorescence intensities of HSA before and after copper(II) complex binding; *K*_*SV*_ is the Stern-Volmer constant; [*Q*] is the molar concentration of the quencher, i.e., metal complex; *k*_*q*_ is the bimolecular quenching rate constant, and *τ*_*o*_ is the lifetime of HSA fluorescence in the absence of any quencher.

2 $$\mathrm{log}\,\frac{({F}_{o}-F)}{F}=\,\mathrm{log}\,{K}_{a}+n\,\mathrm{log}\,[Q]$$

where, *Fo* and *F* are the fluorescence intensities of HSA before and after metal complex binding; *K*_*a*_ is the binding constant; *n* is the number of binding sites, and [*Q*] is the molar concentration of the quencher.

The results presented in Table 1 shows that the *K*_SV_ values (slope of the Stern-Volmer plots) of the three metal complexes were very close and were of the order of 10^5^ thereby indicating a very strong quenching phenomenon. We also calculated the bimolecular quenching constant (*k*_q_) after taking *τ*_o_ of HSA as 5.71 × 10^−9^ s^42^. The *k*_q_ values for the three metal complexes were of the order of 10^13^ M^−1^ s^−1^ which were at least 1000 times more than the maximum collision constant of 10^10^ M^−1^ s^−1^ ^43^. These results clearly indicate that the quenching of HSA fluorescence in the presence of the studied metal complexes was due to the formation of a complex rather than merely a collision event.

**Table 1:** Fluorescence quenching parameters of HSA in the presence of metal complexes.

Complex	*K*_SV_ × 10^5^ (M^−1^)	*k*_q_ × 10^13^ (M^−1^ s^−1^)	*n*	*K*_a_ × 10^6^ (M^−1^)
1	3.786	6.63	1.04	0.60
2	3.689	6.46	1.12	1.52
3	3.663	6.42	1.09	1.11

The slope of the modified Stern-Volmer plot indicates the number of binding sites (*n*) present in the molecule while its intercept gives an idea about the binding constant (*K*_a_). We establish that each of the metal complexes has only one equivalent binding site present on HSA molecule (Table 1). The binding constant of the three metal complexes was of the magnitude of 10^5^–10^6^ M^−1^ which indicated a strong binding of metal complexes with HSA. Earlier studies also indicated that several ligands bind strongly to HSA with a binding constant ranging between 10^3^ M^−1^ to 10^5^ M^−1^ ^44–47^.

#### 3D fluorescence spectroscopy

**Figure 3:**
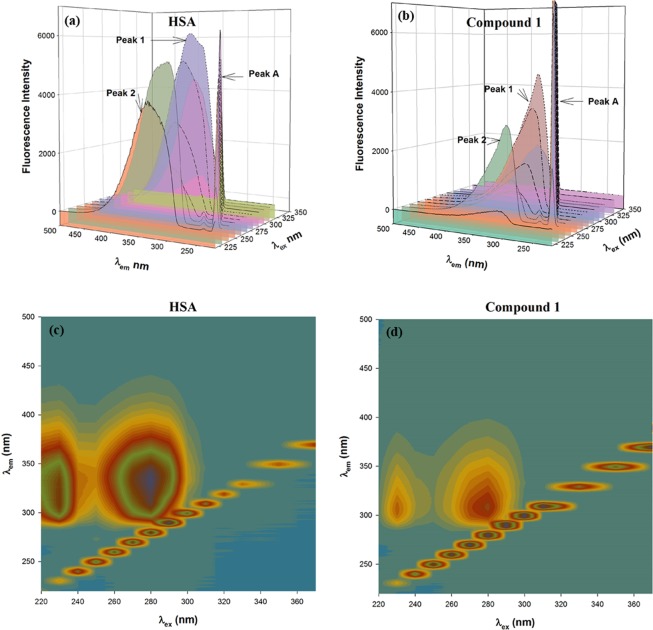
(**a**) Three-dimensional fluorescence of HSA in the presence and absence of metal complexes (**b**) Contour maps depicting three-dimensional fluorescence of HSA in the presence and absence of copper complex **1**.

The conformational changes in HSA upon metal complexes binding was observed by monitoring 3D fluorescence spectra of HSA alone and in the presence of metal complexes in 1:1 molar ratio (Figs 3 and S7). The peak A represented Rayleigh scattering peak (λ_ex_ = λ_em_) while peaks 1 (λ_ex_ = 280 nm) and 2 (λ_ex_ = 230 nm) represented fluorescence spectral features of aromatic amino acid residues due to π → π^*^ transition and polypeptide backbone due to π → π^*^ transition of the C=O bond of HSA. The characteristics of peaks 1 and 2 gave information about the polarity of micro-environment around aromatic amino acid residues and the change in the protein conformation, respectively^48^. Our results indicate that the intensity of peak 1 was decreased by 24–26% while 39–41% reduced that of peak 2 in the presence of metal complexes (Table 2). We also observed a significant blue shift (26–27 nm) in the position of peak 1 implying a great change in the three-dimensional conformation of HSA around the micro-environment of aromatic amino acid residues. On the other hand, a comparatively minor blue shift (4–6 nm) in the position of peak 2 was observed which showed an altered configuration of the peptide backbone also.

**Table 2:** Three-dimensional fluorescence characteristics between HSA and metal complexes.

HSA/Compound	Peak No.	Peak position [λ_ex_/λ_em_ (nm/nm)]	Intensity of the peak	Relative peak intensity
HSA only	1	280/335	6079	100
	2	230/311	5164	100
HSA-Compound **1**	1	280/309	4589	76
	2	230/307	3040	59
HSA-Compound **2**	1	280/309	4581	75
	2	230/306	3084	60
HSA-Compound **3**	1	280/308	4483	74
	2	230/305	3158	61

#### FRET between HSA and Copper (II) complexes

FRET occurs when the emission spectrum of the donor overlaps with the absorption spectrum of the acceptor^49^. Here, we used FRET to measure the distance (*r)* between Trp-214 of HSA and bound metal complex using the following equations:

3 $$E=\frac{{R}_{o}^{6}}{{R}_{o}^{6}+{r}^{6}}=1-\frac{F}{{F}_{o}}$$

where, *E* is the efficiency of the energy transfer; *R*_*o*_ is the distance at which the efficiency of energy transfer becomes 50%; *r* is the distance between HSA (donor) and metal complex (acceptor); *F* and *F*_*o*_ are the fluorescence intensities of HSA in the presence and absence of metal complex (quencher).

Moreover,

4 $${R}_{o}^{6}=8.79\times {10}^{-25}{K}^{2}{n}^{-4}\varphi J$$

where, *K*^2^ is the geometry of dipoles (*K*^2^ = 2/3 for HSA); *n* is the refractive index of the medium (here, it is 1.33); *ϕ* is the fluorescence quantum yield of HSA in the absence of quencher (*ϕ* = 0.118), and *J* is the overlap integral of HSA fluorescence emission spectrum and the absorption spectrum of metal complex.

The following equation determines the overlap integral (J)

5 $$J=\frac{{\int }_{0}^{\infty }{F}_{\lambda }{\varepsilon }_{\lambda }{\lambda }^{4}d\lambda }{{\int }_{0}^{\infty }{F}_{\lambda }d\lambda }$$

where, *F*_*λ*_ is the fluorescence intensity of HSA at the wavelength *λ*; *ε*_*λ*_ is the molar extinction coefficient of the metal complex at the wavelength *λ*.

**Figure 4:**
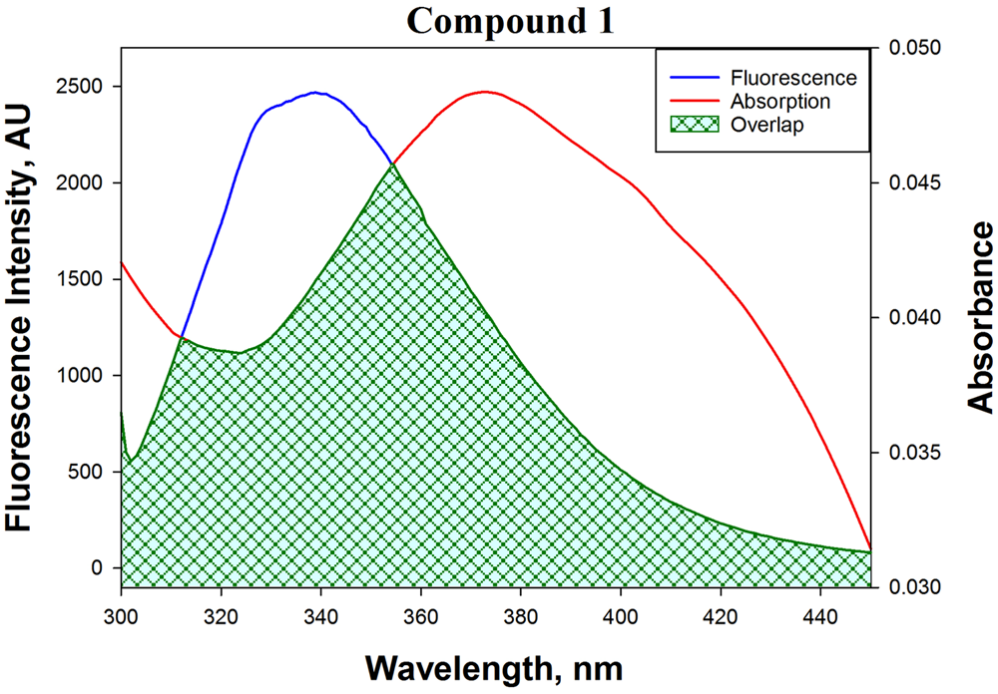
FRET between HSA and copper complex **1**.

Figure 4 (see SI, Fig. S8) depicts the overlap between the HSA fluorescence spectrum and the absorption spectrum of the metal complex and the deduced parameters are presented in Table 3. We found that the efficiency of energy transfer between HSA and metal complexes were in the range of 25.1–38.4%. The value of *r* for copper complexes **1**, **2** and **3** was 3.39 nm, 3.44 nm, and 2.98 nm, respectively. The deduced *r* values satisfied the condition of FRET that it should be within 2–8 nm range. Moreover, the *r* values were within the range of 0.5 *R*_*o*_ < *r* < 1.5 *R*_*o*_ thereby indicating that the quenching of HSA fluorescence was due to a complex formation between HSA and metal complexes (i.e., static quenching mechanism).

**Table 3:** FRET parameters for HSA-metal complex system.

Compound	*J* (M^−1^ cm^3^)	*Ro* (nm)	*r* (nm)	*E* (%)
1	3.45	3.02	3.39	33.2
2	2.51	2.87	3.44	25.1
3	1.99	2.76	2.98	38.4

### Molecular Modelling Studies

#### Prediction of binding sites of metal complexes on HSA

**Figure 5:**
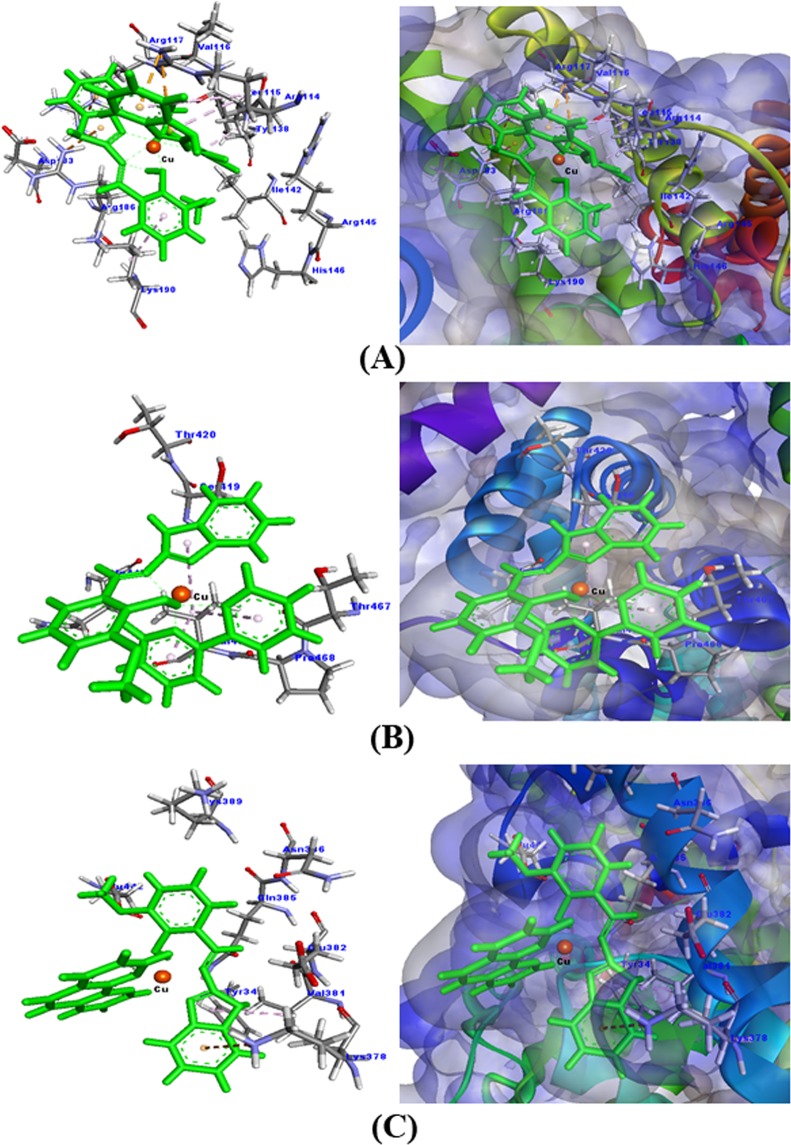
Molecular docking of HSA with different metal complexes. (**A**) HSA-**1**, (**B**) HSA-**2**, and (**C**) HSA-**3** complex.

HSA, the most plentiful protein in the plasma which is responsible for the transportation of drugs and other molecules from one place to another. The primary sequence of HSA folds into three independent domains (I–1III). The domain I span residues 1–195, domain II extends from residues 196–383 and domain III extends from residues 384–585. Each domain has been divided into two subdomains namely A and B^41^. The two principal binding sites of HSA are located in the hydrophobic cavities of subdomain IIA (Sudlow’s site I) and subdomain IIIA (Sudlow’s site II). A new binding site on HSA which is located at subdomain IB has been recently identified^50^. The binding site of metal complexes on HSA was predicted by *in silico* approach using Hex 8.0.0 (Fig. 5; Table 4). The docking scores for the three metal complexes were −339.93, −238.11 and −221.07, respectively indicating a good binding. In Fig. 5, It is clear that the three copper(II) complexes were bound near Trp214 which explains the quenching of HSA fluorescence in the presence of these copper (II) complexes.

**Table 4:** Molecular interactions of HSA with metal complexes.

Metal complex	Docking score	No. of interactions	Interaction	Nature of interaction	Distance (Å)
Complex 1	−336.93	10	Unk:H-Leu115:O	Hydrogen bond	2.08
			Arg114:NH1-Unk	Electrostatic (π-cation)	4.17
			Arg114:NH1-Unk	Electrostatic (π-cation)	3.19
			Arg117:NH2-Unk	Electrostatic (π-cation)	4.55
			Arg186:NH1-Unk	Electrostatic (π-cation)	3.35
			Arg114-Unk	Hydrophobic (π-alkyl)	4.18
			Unk-Leu115	Hydrophobic (π-alkyl)	3.99
			Unk-Arg114	Hydrophobic (π-alkyl)	5.34
			Unk-Lys190	Hydrophobic (π-alkyl)	4.21
			Unk-Arg117	Hydrophobic (π-alkyl)	4.81
Complex 2	−238.11	3	Val469-Unk	Hydrophobic (π-alkyl)	4.58
			Unk-Val469	Hydrophobic (π-alkyl)	5.04
			Unk-Val469	Hydrophobic (π-alkyl)	3.97
Complex 3	−221.07	3	Unk:H-Glu442:OE1	Hydrogen bond	3.09
			Lys378: NZ-Unk	Electrostatic (π-cation)	4.89
			Val381-Unk	Hydrophobic (π-alkyl)	4.85

### *In vitro* Anticancer Activity

#### Cytotoxicity assessment (MTT Assay)

The cytotoxicity of all the three copper(II) complexes (1–3) were tested on cultured MCF-7 (human breast cancer) cell lines by exposing for 24 h to the medium containing the complex concentrations 1–100 µM in comparison with the widely used drug cisplatin under identical conditions by MTT assay. A concentration dependent cytotoxic response was observed after the exposure of different concentrations of the complexes. Complex 3 was found to be more cytotoxic followed by complex 2 and complex 1.

**Figure 6:**
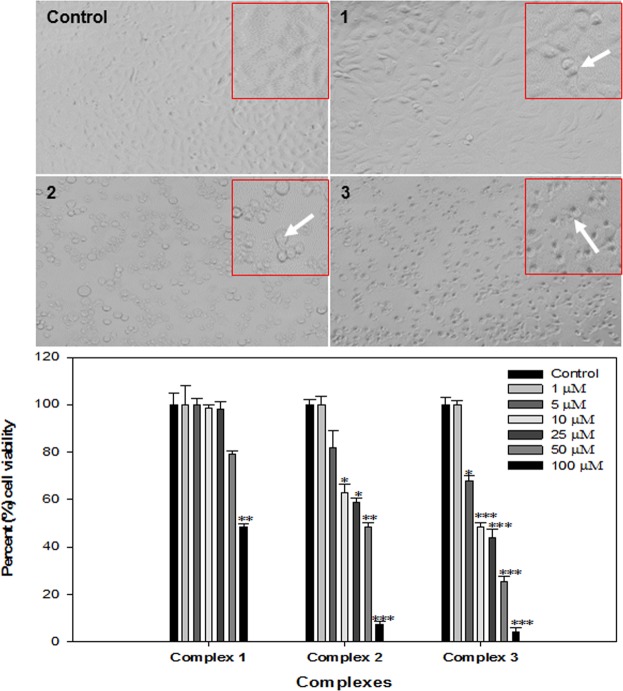
Cytotoxicity assessment by MTT assay and morphological changes induced by copper complexes in MCF7 cells. Cells were exposed to different concentrations of copper complexes for 24 h. Morphological images were grabbed using a phase contrast microscope at 20x magnifications. White arrows in the images showing the apoptotic cells. *p < 0.05, **p < 0.005, ***p < 0.001 vs control.

The results of the cytotoxic activities on MCF-7 cell lines was determined according to the dose values of potential drug exposure required to reduce survival in the cell lines to 50% (IC_50_). The concentration-dependent percentage cell viability histogram is given in Fig. 6, from which the IC_50_ value can be deducted.

As it is marked from the histogram, with the increasing concentration of copper(II) complexes was accompanied by a progressive decrease in the percentage cell viability. Upon increasing the concentration of complex the adsorption of the complex on cell membranes increases, leading to increasing penetration, facilitates binding with DNA bases and causes efficient cell killing activity. The copper complexes **1**–**3** displayed a concentration-dependent cytotoxic profile in MCF-7 cell lines. Since the IC_50_ values for complexes **1**–**3** were statistically lower than that for metal-free Schiff base (L) and heterocyclic moieties viz. Bpy and Phen in the tested cells, it recommended that Cu(II) ion play a key role in facilitating complexes potency. It was evident that complex **3** exhibited higher cytotoxicity than the other two complexes against MCF-7 cell lines. The cytotoxic activity of complex **3** can be due to stronger DNA binding ability of the planar “phen” co-ligand by deeper insertion between the DNA base pairs, consequently leading to cell death. The morphology examinations also showed that the proliferation of the cells was significantly inhibited and the cells exhibit morphological changes such as cell shrinkage and cell detachment, as shown in Fig. 6.

The cytotoxicity’s of the copper(II) complexes **1**–**3** on normal human embryonic kidney (HEK293) cell line were also studied. These copper(II) complexes exhibited very low cytotoxicity in HEK293 cell line at the tested concentrations as compared to the MCF-7 cells (see SI, Fig. S9).

### Copper complexes inducing Apoptosis (Annexin V-FITC/PI staining)

Induction of the apoptosis by the copper(II) complexes **1**–**3** against MCF-7 cancer cell line was analyzed by the help of the Annexin V-FTIC/PI double staining assay. The results are presented in the dot plots in Fig. S10 (see SI). The level of phosphatidylserine quantified the treatment of the MCF-7 cells with the complexes **1**–**3** for 24 h exhibited results as viable, early apoptosis, late apoptosis and necrosis, and their percentage. All the three complexes **1**–**3** exhibited an increase in apoptotic cells to a significant level followed by necrosis and finally, loss of plasma integrity. Thus, these results confirm that copper(II) complexes **1**–**3** induce apoptosis in MCF-7 cancer cell lines. However, the level of the apoptosis is more in complex **3** as compared **1** and **2**.

#### Intracellular Glutathione (GSH) depletion

GSH stands out as a main and integral oxidant scavenger in all mammalian cell types. GSH redox status is very critical for various biological processes, viz. transcription activation of specific genes, regulation of redox-related signal transduction pathways, and control of cell proliferation and apoptosis^51,52^. Recent studies have marked the significance of intracellular GSH in the anticancer therapy. Due to the cytotoxicity of antitumor drugs depend significantly on intracellular levels of GSH. The GSH depletion can facilitate ROS accumulation and potentiate the lethality in antitumor drug-treated cells^53–55^.

**Figure 7:**
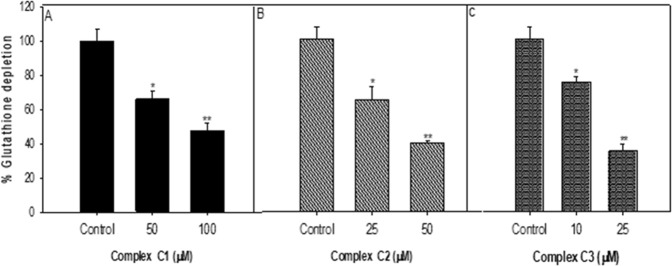
Percent change in glutathione level in MCF-7 cells exposed to copper complexes for 24 h. *p < 0.05, **p < 0.005, ***p < 0.001 vs control. The results of depletion in the glutathione level in cultured MCF-7 cells exposed to Cu(II) complexes (**1**–**3**) for 24 h are summarized in Fig. 7. The results confirm a significant decrease in GSH level and were found the maximum in case of complex 3 to 60–80% at a lower concentration of 10 and 25 µM, respectively in MCF-7 cells as compared to control. Whereas the other two complexes **1** (50 and 100 µM) and **2** (25 and 50 µM) have shown significant depletion of GSH level but at quite higher concentration.

Cells with reduced cellular GSH levels should be more sensitive to the effects of Cu(II) complex if oxidative stress has a role in their cytotoxicity. In cancer cells, the GSH/GSSG ratio has been shown to influence the regulation of the cell cycle, mutagenic mechanisms, DNA synthesis, growth, and multidrug and radiation resistance, and GSH levels are typically higher in tumor tissue than in normal tissue^40,56–60^. The GSH level of cells that had been exposed to copper(II) complexes **1**–**3** was determined (Fig. 7).

#### Lipid peroxidation

Lipid peroxidation (LPO), a natural process in the cellular system and is one of the most studied consequences of reactive oxygen species (ROS) generation in cell membrane structure and function. It is also known that lipid hydroperoxides and oxygenated products of lipid degradation, as well as initiators (i.e., ROS), can contribute to the signal transduction cascade^58–60^, cell proliferation, differentiation, maturation, and apoptosis^61–63^. Therefore, in this study, we have observed the value of LPO in MCF-7 cells exposed to copper complexes using the TBARS method. A concentration-dependent substantial increase in LPO level was observed in MCF-7 cells treated with different concentrations of Cu(II) complexes **1**–**3** for 24 h. The results showed that the increase in lipid peroxidation was significantly higher in complex **3** exposed MCF-7 cells as compared to complex **1** and **2** which correlates well with the other studies.

These findings suggest that an increase in lipid peroxidation in MCF-7 cells is because of the generation of ROS (Fig. S11, see SI). These findings can also be explained by considering the redox changes that are promoted by Cu(II) compounds inside the cells. Cu(II) complex, which has a higher redox potential and generates larger amounts of the oxidative hydroxyl radical (OH^•^, E° = 2.3 V); thus, the observed oxidation of lipid molecules is stimulated^64^. In this context, polyunsaturated membrane lipids are particularly susceptible to peroxidation by the readily oxidizable bis-allylic hydrogens (redox couple E° = 0.60 V), which allow facile initiation of the oxidative chain reaction.

**Figure 8:**
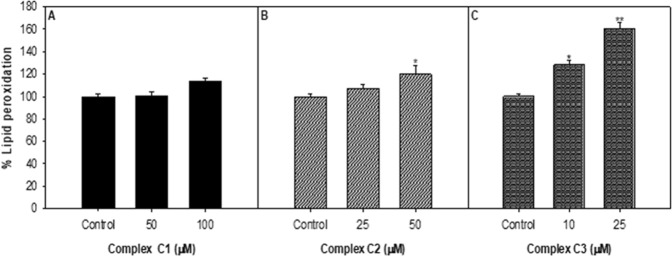
Percentage change in lipid peroxidation in MCF-7 cells exposed to copper complexes for 24 h. *p < 0.05, **p < 0.005, ***p < 0.001 vs control.

Propagation of this reaction is thermodynamically and kinetically favored because the lipids react rapidly with peroxyl radicals (HO_2_^•^) while the lipid radicals interact with superoxide (O_2_^−•^). Cu(II) complex induced lipid peroxidation in MCF-7 cells exposed for 24 h are abridged in Fig. 8. A concentration-dependent significant increase in the lipid peroxidation was observed.

### *In-Vivo* NSAIDs Studies

The inflammatory process involves edema, pain, tenderness, and redness. These symptoms evolve as a result of the production of various chemical mediators namely; Leukotrienes, histamine, HETEs, and prostaglandins. By the hydrolytic effect of the enzyme phospholipase, A2, Arachidonic acid is liberated from cell membrane phospholipids. Subsequently, arachidonic acid is hydrolyzed to prostaglandins (by the action of cyclooxygenases enzymes) and leukotrienes (by the action of lipoxygenases enzymes)^65^. Prostaglandins are the mediators of pain along with vasodilation and capillary permeability. Substances that relieve pain and/or decreases body temperature most probably work by either inhibition of phospholipase A_2_ or cyclooxygenases. On the other hand, leukotrienes (LTs) are responsible for capillary permeability, chemotaxis of inflammatory mediators and extravasation of white blood cells leading to sustaining of inflammation^66^. Substances that inhibit inflammation alone most probably work by inhibiting lipoxygenases. Generally, prostaglandins are the major mediators of hyperpyrexia, and pain sensitization and the LTs are the primary mediators of inflammation development^67^. Substances that inhibit inflammation, pain and decrease body temperature most probably work by inhibiting phospholipase A_2_ alone or with lipoxygenases and cyclooxygenases enzymes^68^.

**Figure 9:**
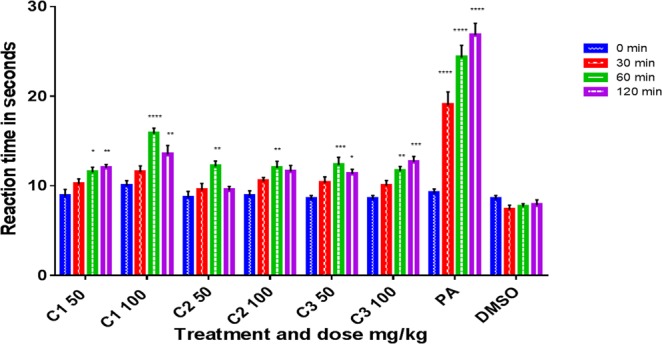
Effects of C1 (**1**), C2 (**2**), and C3 (**3**) on acetic acid-induced pain (algesia). Results are presented as mean ± SEM, n = 6. Readings for each group were compared with pretreatment (0 min-the control) reading. Paracetamol (PA) was used as standard (positive control) and dimethyl sulphoxide (DMSO) as vehicle control. ****p < 0.001, ***p < 0.005, *p < 0.05, ANOVA with Dunnett’s as post hoc test N = 6.

**Figure 10:**
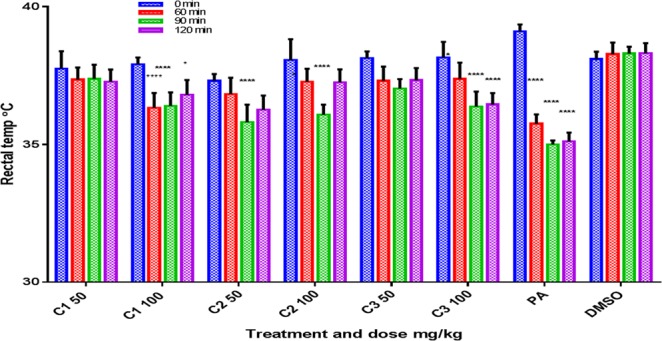
Effects of C1 (1), C2 (2), and C3 (3) on yeast-induced hyperthermia. Results are presented as mean ± SEM. Readings for each group were compared with pretreatment (0 min-the control) reading. Paracetamol (PA) was used as standard (positive control) and dimethyl sulphoxide (DMSO) as vehicle control. ****p < 0.001, *p < 0.05, ANOVA with Tukey’s as post hoc test N = 6.

**Figure 11:**
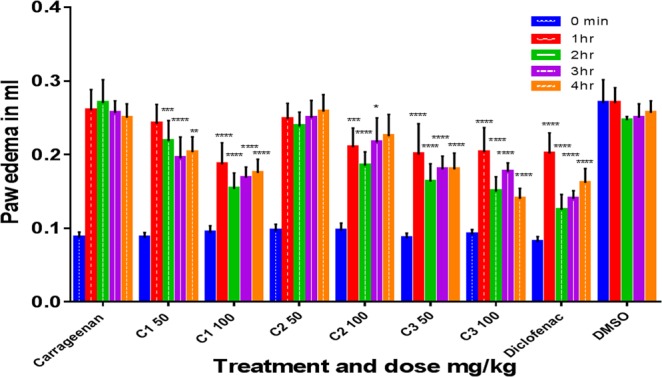
Effect of different doses of C1, C2 and C3 on carrageenan-induced paw edema in rats. Results are presented in mean ± SD compared with carrageenan group (control group), n = 6. Diclofenac was used as standard (positive control) and dimethyl sulphoxide (DMSO) as vehicle control. *p < 0.05, **p < 0.01, ***p < 0.005, ****p < 0.001, ANOVA with dunnett’s as post Hoc test N = 6.

C1 (complex **1**) and C3 (complex **3**) produced significant dose-dependent and potent analgesic and anti-inflammatory effects and significant antipyretic activity only at 100 mg/kg (*p* < 0.05, n = 6, Figs 9–11). The two activities (analgesic and anti-inflammatory) of the two compounds might be accomplished by inhibiting both LTs and prostaglandins which could be achieved either by inhibiting both cyclooxygenase and lipoxygenases enzyme families or by inhibiting phospholipase A2. C2 (complex **2**) may not have a strong role on phospholipase A2 or lipoxygenases since it produced significant and dose-dependent antipyretic activity (*p* < 0.05, n = 6) but only short significant anti-inflammatory and analgesic effects at 100 mg/kg (*p* < 0.005, n = 6). The weak anti-inflammatory and analgesic activities most probably exclude at least partly the involvement of lipoxygenases and phospholipase A2 enzymes and may involve inhibition of only cyclooxygenases enzymes.

**1** and **3** decrease pain significantly starting from 60 min and up to 120 min in a dose-dependent way (*p* < *0*.*05*, n = 6, Fig. 9). 2 decreased pain significantly only at 60 min. However, it is short acting since no significant inhibition was noticed at 120 min.

**1** and **3** did not decrease body temperature at 50 mg/kg (the significant transient effect was noticed for C3 at 90 min only). **1** and **3** at 100 mg/kg produced a potent and significant decrease in body temperature till 120 min (*p* < 0.05, n = 6, Fig. 10). **2** at a dose of 50 mg/kg produced a significant decrease in body temperature whereas at 100 mg/kg produced a significant transient effect at 90 min (*p* < 0.05, n = 6, Fig. 10).

No statistically significant difference was noticed between the inflammation reading at 0 time in the carrageenan group and the inflammation readings at 0 times in all the other groups.

**1** and **3** produced potent significant and dose-dependent anti-inflammatory effects which last for up to 4 h after carrageenan injection (*p* < 0.001, n = 6, Fig. 11). On the other hand, **2** produced significant and short anti-inflammatory effect only at 100 mg/kg which stops after 3 h from carrageenan injection.

#### In silico Prediction of binding sites of copper complexes on COX-2

**Figure 12:**
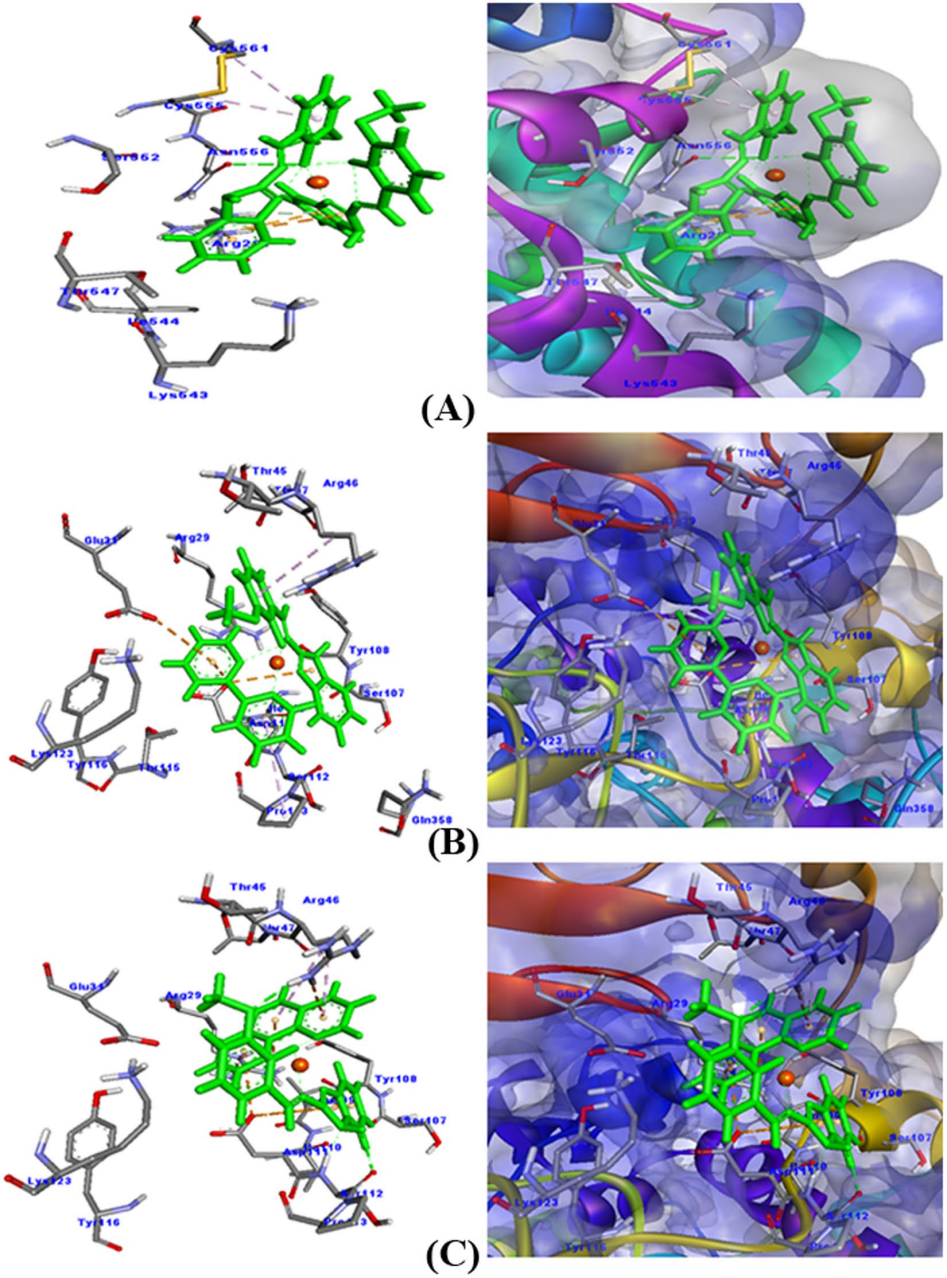
Molecular docking of COX-2 with different copper(II) complexes.

COX-2 is an enzyme, which is responsible for the formation of prostanoids and prostaglandins. Drugs that specifically inhibit COX-2 enzyme have been found to provide relieve from pain and inflammation. In this study, the potential of copper complexes **1**–**3** has been studied to act as an inhibitor of COX-2 though molecular docking studies. The *in silico* approach empowers us to visualize the specific binding of copper(II) complexes with COX-2 and the molecular interactions that play a significant role in stabilizing this binding. The result presented in Fig. 12 and Table 5 indicates that all the three copper(II) complexes were bound strongly to COX-2. The docking score of the tested compounds were −262.98, −374.96 and −327.67 for copper(II) complexes **1**, **2** and **3** respectively. It is worth to note that **1** formed 7 molecular interactions with COX-2. These were hydrogen bonds formations with Asn556 (2.80 Å), Arg297 (2.75 Å), electrostatic interactions (π-cation) with Arg297 and hydrophobic interactions (π-alkyl) with Cys556 and Cys561. Similarly, **2** formed 6 bonds with COX-2, namely hydrogen bond with Arg46 (2.38 Å), electrostatic interactions (π-anion) with Glu31 and Asp111, and hydrophobic interactions (π-alkyl) with Arg46 and Pro113. The maximum number of interactions was formed between **3** and COX-2 as illustrated in Fig. 12. The copper(II) complex formed one hydrogen bond with Arg46 (2.08 Å) and two hydrogen bonds with Ser112 (2.18 Å and 2.36 Å). It also interacted with COX-2 through six electrostatic interactions (π-cation) with Arg29, Arg46, and Asp111. Moreover, **3** and COX-2 binding was also stabilized by two hydrophobic interactions (π-alkyl) with Arg46.

**Table 5:** Molecular interactions of COX2 with copper(II) complexes.

Copper(II) complex	Docking score	No. of interactions	Interaction	Nature of interaction	Distance (Å)
Complex 1	−262.98	7	Unk:O-Asn556:OD1	Hydrogen bond	2.80
			Arg297: HE-Unk	Hydrogen bond	2.75
			Arg297:NH2-Unk	Electrostatic (π-cation)	3.69
			Arg297:NH2-Unk	Electrostatic (π-cation)	4.62
			Arg297:NH2-Unk	Electrostatic + Hydrogen bond (π-cation; π –donor	4.18
			Unk: O-Cys555	Hydrophobic (π-alkyl)	4.42
			Unk: O-Cys561	Hydrophobic (π-alkyl)	5.02
Complex 2	−374.96	6	Arg46:HH12-Unk:O	Hydrogen bond	2.38
			Glu31: OE2-Unk	Electrostatic (π-anion)	4.46
			Asp111:OD1-Unk	Electrostatic (π-anion)	4.66
			Asp111:OD1-Unk	Electrostatic (π-anion)	2.96
			Unk-Arg46	Hydrophobic (π-alkyl)	4.88
			Unk-Pro113	Hydrophobic (π-alkyl)	5.12
Complex3	−327.67	11	Arg46:HH12-Unk:O	Hydrogen bond	2.08
			Ser112:HN-Unk: N	Hydrogen bond	2.18
			Unk: H-Ser112:O	Hydrogen bond	2.36
			Arg29:NH1-Unk	Electrostatic (π-cation)	3.83
			Arg29:NH2-Unk	Electrostatic (π-cation)	4.95
			Arg46:NH1-Unk	Electrostatic (π-cation)	4.39
			Asp111:OD1-Unk	Electrostatic (π-anion)	4.78
			Asp111:OD1-Unk	Electrostatic (π-anion)	4.96
			Asp111:OD1-Unk	Electrostatic (π-anion)	4.26
			Unk-Arg46	Hydrophobic (π-alkyl)	4.34
			Unk-Arg46	Hydrophobic (π-alkyl)	5.47

## Conclusion

Herein, we have designed, synthesized and characterized three copper complexes **1**–**3** of biocompatible Schiff base ligand “2-[(1H-benzoimidazol-2-ylimino)-methyl]-6-methoxy-phenol” promising entities for target-selective next-generation anticancer and NSAIDS therapeutics. All the three complexes **1**–**3** were tested for it a biological application, starting from the interaction of complexes **1**–**3** with model protein HSA, using *In vitro* fluorescence technique and *in silico* molecular modeling studies, which exhibited strong binding propensity. Further, the complexes **1**–**3** were tested for *In vitro* cytotoxicity against MCF-7 human breast cancer cell lines; the results showed the profound potential of **3** in comparison to **1** and **2**. Moreover, to know the mechanism of action of the complexes the lipid peroxidation and glutathione depletion was also studied which revealed significant role of the ROS generation in the cytotoxicity. The morphological images of the MCF-7 cancer cells, upon treatment with complexes **1**–**3** displayed nuclear blebbing and fragmentation of the nuclei, which is typical of late, apoptosis. Later on, these complexes were tested *in vivo* on albino rats and mice for anti-inflammatory, antipyretic and analgesic activities; the results showed **1** and **3** significant dose-dependent anti-inflammatory and analgesic activities at a lower concentration. Besides, we have carried out the *in silico* study of complexes **1**–**3** with a COX2 inhibitor which confirmed the interaction of complexes with COX2 inhibitor, which can be a mechanism of action of these potential candidates for NSAIDS. Therefore complexes **1** and **3** are promising candidates to act as anticancer and COX 2 inhibitor (NSAIDs) agents, thus warrant further detailed investigations *in vivo*.

## Experimental

### Material and Methods

2-Aminobenzimidazole, o-vanillin, Cu(NO_3_)_2_∙3H_2_O, 1,10-phenanthroline, 2,2′-bipyridyl, Acetic acid, All the solvents were used as purchased from the company Sigma-Aldrich without further purification. Infrared spectra were recorded as KBr pellets, using a Shimadzu IRAffinity-1 spectrometer with a resolution of 4 cm^−1^. Elemental analysis (C, H, N) were performed on a PerkinElmer 2400 Series II CHNS/O system. NMR spectra were recorded on JEOL-ECP-400 spectrometer. The EPR spectrum of the copper complex was acquired on a Varian E 112 spectrometer using X-band frequency (9.1 GHz) at liquid nitrogen temperature in the solid state. Fatty acid-free HSA (99% pure) was purchased from Sigma and used without further purification. Methanol was of HPLC grade and obtained from Fisher scientific UK. Other reagents, sodium acetate anhydrous (Win lab, UK) and acetic acid (Riedel-de Haen Germany) were used. The mobile phase pH was recorded using a pH meter (model TS 625, Thomas Scientific, USA). Purified water was prepared using a Millipore Milli-Q (Bedford, MA, USA) water purification system. We have used DMSO (<1%) as the solvent to dissolve the complexes.

All the animal-based experiments were performed in compliance with the relevant laws and institutional guidelines and had been approved and permitted by the ethics committee (Letter No. CBR 4537) of the College of Pharmacy, King Saud University, Riyadh, KSA. We used DMSO (<1%) in the *In vitro* and animal studies as well.

### Synthesis of Schiff base (L)

The ligand was synthesized as reported earlier by some of us^34^. In brief, the equimolar amounts of vanillin (0.354 g, 2.0 mmol) and 2-aminobenzimidazole (0.266 g, 2.0 mmol) and in absolute ethanol (10 ml) with few drops of acetic acid were refluxed, filtered and washed.

#### Synthesis of Copper complex [Cu(BImSB)_2_] (1)

This has been synthesized by adopting a similar method as reported by some of us^34^, in brief, A Schiff base (0.267 g, 1.0 mmol) methanolic solution (5 ml) was added slowly to a methanolic solution (10 ml) of Cu(NO_3_)_2_⋅3H_2_O (0.120 g, 0.5 mmol) and stirred for 4 h. Then, the mixture was filtered and washed and dried.

#### General synthetic procedure

Complexes **2** and **3** were prepared by a general synthetic procedure in which a 0.241 g (1.0 mmol) quantity of copper(II) nitrate.trihydrate in 15 mL aqueous methanol (1:1 v/v) was reacted with the heterocyclic base (L: bpy, 0.15 g, 1.0 mmol and phen, 0.19 g, 1.0 mmol) while stirring at room temperature for 0.5 h followed by addition of methanolic solution of Schiff base ligand “L” (1.0 mmol, 0.267 g) in small portions with continuous stirring. The reaction mixture was stirred for 4 h, and the product was isolated as a green solid in 77–80% yield. The product isolated is washed with water and cold methanol followed by drying in vacuum.

#### Synthesis of Copper complex [Cu(BImSB)(bpy)]NO_3_ (2)

Yield: 510 mg, 77%. Anal. Calcd. for (C_25_H_22_N_6_O_5_Cu) (549.1): C, 54.59; H, 4.03; N, 15.28. found: C, 54.39; H, 4.04; N, 15.27. ESI–MS *m/z* {in DMSO, observed (calcd)} for [Mw – NO_3_ + H^+^] 486.2, (485.1). IR (KBr disc): 3429br, 3336br, 1690vs, 1610vs, 1541 m, 1519w, 1479w, 1428 m, 1384 m, 1342br, 1246 m, 1212 s, 848 m, 740 s, 721 s, 650w. μ_eff_ = 1.83 BM.

#### Synthesis of Copper complex [Cu(BImSB)(phen)]NO_3_ (3)

Yield: 535 mg, 80%. Anal. Calcd. for (C_27_H_20_N_6_O_5_Cu) (571.09): C, 56.69; H, 3.52; N, 14.69. found: C, 56.63; H, 3.52; N, 14.57. ESI–MS *m/z* {in DMSO, observed (calcd)} for [Mw – NO_3_ + H^+^] 510.1, (508.1). IR (KBr disc): 3424br, 3334br, 1697vs, 1608vs, 1542 m, 1519w, 1479w, 1418 m,1383 s, 1346 m, 1248 m, 1212 s, 845 m, 768 m, 740 s, 721 s, 649w. μ_eff_ = 1.79 BM.

### *In vitro* Anticancer Activity

#### Cell culture

MCF-7 cells were grown in Dulbecco’s modified eagle’s medium (DMEM) supplemented with 0.2% sodium bicarbonate, 10% fetal bovine serum (FBS), and antibiotic/antimycotic solution (100X, 1 ml/100 ml of medium). The cells were maintained in 5% CO_2_–95% atmosphere under high humidity at 37 °C. Cells were assessed for cell viability by trypan blue dye exclusion assay as described earlier^69^. The batches of cells showing more than 98% of cell viability were used in this study.

#### Cytotoxicity by MTT assay

Percent cell viability was assessed using the MTT assay as described before^70^. Briefly, 10,000 cells were plated in 96 well culture plates and allowed to adhere for 24 h in a CO_2_ incubator at 37 °C. After the exposure, MTT (5 mg/ml of stock in PBS) was added (10 μl/well of 100 μl of cell suspension) in each well and plates were incubated further for 4 h in a CO_2_ incubator. Then, the supernatant was discarded, and 200 μl of DMSO was added to each well and were mixed gently. The developed color was read at 550 nm. Untreated control sets were also run under identical conditions. All the values were corrected from background absorbance.

#### Morphological analysis by phase contrast microscope

Morphological changes were observed to determine the alterations induced by copper complexes in MCF-7 cells. All the cells were exposed to different concentrations of copper complexes for 24 h. The cell images were acquired at 20x magnification under the phase contrast inverted microscope.

#### Glutathione (GSH) level

The depletion in GSH level was estimated following the protocol of Chandra *et al*.^71^. In brief, after the exposure, cells were collected by centrifugation, and the cellular protein was precipitated by incubating 1 ml of the sonicated cell suspension with 1 ml TCA (10%) and was placed on ice for 1 h and then by centrifugation for 10 min at 3000 rpm. The supernatant was added to 2 ml of 0.4 M Tris buffer (pH 8.9) containing 0.02 M EDTA, followed by the addition of 0.01 M 5,5′-dithionitrobenzoic acid (DTNB) to a final volume of 3 ml. At 37 °C, for 10 min of incubation in a shaking water bath, the tubes were kept. The absorbance of the yellow color developed was read at 412 nm.

#### ROS generation

Intracellular ROS generation was measured using DCFH-DA fluorescent dye following the protocol of Al-Sheddi *et al*.^72^. In brief, after the exposure of MCF-7 with copper complexes, the cells were washed twice with PBS. Then the cells were exposed to 20 µM of DCFH-DA for 1 h in dark at 37 °C. The fluorescence intensity of dye was measured at 485 nm excitation and 530 nm emission wavelengths using spectrofluorometer.

#### Lipid peroxidation (LPO)

Lipid peroxidation was performed using protocol “thiobarbituric acid-reactive substances” (TBARS)^73^. Briefly, after the exposure, cells were collected by centrifugation and were sonicated in ice-cold KCl solution (1.15%) and were then centrifuged for 10 min at 3000xg. The resulting supernatant (1 ml) was added to 2 ml of thiobarbituric acid (TBA) reagent (15% TCA, 0.7% TBA and 0.25 N HCl) and was heated at 100 °C for 15 min in a boiling bath. Then samples were placed in the cold and were centrifuged at 1000 × g for 10 min. At 550 nm, the absorbance of the supernatant was measured.

### *In Vivo* Biological Studies

#### *Anti-inflammatory activity* (Carrageenan-induced paw edema in rats)

Pedal inflammation in albino rats (8 to10 weeks old) of either sex weighing 180–200 g was produced according to the method described by Winter *et al*.^65^. An injection was made of 0.0 5 ml of 1% carrageenan sodium salt (BDH) into the right hind foot of each rat under the plantar aponeurosis. The test groups of rats were treated orally with 50 and 100 mg/kg 1 h before the carrageenan injection. At the same time, the control group was given 5 ml/kg of normal saline, and the reference group was given 100 mg/kg of an aqueous solution of oxyphenbutazone. The measurements of foot volume were done by the displacement technique using a plethysmometer (Apelex, France) immediately after and +2 and +3 h after the injection of carrageenan. The inhibitory activity was calculated according to the following formula:

$${\rm{Percent}}\,{\rm{inhibition}}=100[1-({\rm{a}}-{\rm{x}})/({\rm{b}}-{\rm{y}})]$$

Where ‘b’ is the mean paw volume of control rats after carrageenan injection and ‘y’ before the injection; whereas ‘x’ is the mean paw volume of treated rats before injection and ‘a’ is the mean paw volume after carrageenan injection.

#### *Antipyretic test* (yeast induced hyperpyrexia in mice)

Hyperpyrexia was induced in mice by subcutaneous injection of (20% aqueous suspension of brewer’s yeast) of 20 ml/kg body weight (6 animals in each group) in the back below the nape of the neck (Loux *et al*.)^66^. The animals then fasted for the duration of the experiment (approximately 26 h); water was made available *ad lib*. Control temperatures were taken 24 h after the yeast injection to determine the pyretic response to yeast. Rectal temperatures taken 1 h before drug administration in fevered animals served as a pre-drug control.

### *Analgesic Activity* (Acetic acid induced writhing test)

Acetic acid-induced writhing in the mice-The test was carried out using the technique of Siegmund *et al*.^67^, as modified by Koster *et al*.^68^. Both the complexes (50 and 100 mg/kg body weight) were administered orally, to 16 h fasten mice, and each group of six animals was divided. Later, upon 1 h of treatment, the mice were injected intraperitoneally with 0.2 ml of 3% acetic acid solution to induce the characteristic writhings. The number of writhing’s occurring between 5 and 15 min after the acetic acid injection in control, and treated animals were recorded. The responses of compounds-treated groups were compared with those of animals receiving in diclofenac sodium (as a standard drug), 4 mg/kg, as well as with the control group.

### Fluorescence quenching measurements

The samples for measuring quenching in fluorescence of HSA in the presence of copper(II) complexes were prepared in 20 mM sodium phosphate buffer (pH 7.4). The concentration of HSA was determined from Beer-Lambert’s law using molar extinction coefficient of 36500 M^−1^ cm^−1^ at 280 nm^74^. The stock solution (1 mM) of copper(II) complexes were prepared in DMSO and further diluted in sodium phosphate buffer until the desired concentration is reached. The final DMSO concentration was less than 1%. The fluorescence measurements were performed on JASCO spectrofluorometer (FP-8300) fitted with a thermostatically controlled cell holder attached to a water bath.

The quenching of fluorescence of HSA was monitored by exciting the fluorophore at 295 nm and measuring spectrum in the range 300–450 nm^35,75^. The excitation, as well as emission slits, was kept at 5 nm. All the fluorescence intensities were corrected for the inner filter effect as explained in the results and discussion. The 3D fluorescence was measured by monitoring the emission spectrum of HSA alone or in combination with metal complexes in 220–700 nm range. The fluorophore was excited at 220 nm with a successive increment of 10 nm. The excitation and emission slits were set at 5 nm. FRET between HSA and metal complexes was observed by measuring absorption spectra of metal complexes in the 300–450 nm range where HSA emits its fluorescence.

### *In silico* interaction studies

The three-dimensional coordinates of HSA (PDB ID: 1H9Z) and COX-2 (PDB ID: 3LN1) were downloaded from the RCSB Protein Data Bank.CHEMSKETCH (http://www.acdlabs.com) was used to draw the structures of copper(II) complexes. The.mol format was converted into.pdb format using OPENBABEL (http://www.vcclab.org/lab/babel). The ligand and receptor files were prepared for molecular docking by removing water molecules and any hetero-atoms as described previously^76^. The ligand and receptor files were energy-minimized using Discovery Studio 4.0 (Accelrys Software Inc., 2013)^77^. HEX 8.0.0 software was used to study the interaction between proteins (HSA and COX-2) with copper(II) complexes. We set the correlation type to Shape + Electro, and OPLS minimization did the post-processing. The GRID dimension was set at 0.6, and 10000 solutions were computed. Other parameters were used as defaults. The docking figures for publication were prepared in Discovery Studio 4.0 (Accelrys Software Inc., 2013)

### Statistical analysis

Results are expressed as mean ± SEM. One-way ANOVA was used for a comparison test of significant differences among groups followed by Dunnet’s multiple comparison posts- tests. A level of significance (P < 0.05) was considered for each test.

## Supplementary information

supplemetary info
