# Supplementary material for: Copper(II) complexes as potential anticancer and Nonsteroidal anti-inflammatory agents: In vitro and in vivo studies
Source: Sci Rep. 2019 Mar 27;9:5237. doi: 10.1038/s41598-019-41063-x (PMC6437194; doi:10.1038/s41598-019-41063-x)
Supplement: Supplementary file 1 — supplemetary info [file 41598_2019_41063_MOESM1_ESM.doc]

**Supplementary Data**

**Copper(II) complexes as potential anticancer and Nonsteroidal anti-inflammatory agents: *in vitro and in vivo studies***

Afzal Hussain,^a^ Mohamed Fahad AlAjmi,^a^ Md. Tabish Rehman,^a^ Samira Amir,^b^ Fohad Mabood Husain,^c^ Ali Alsalme,^d^ Maqsood Ahmad Siddiqui,^e^ Abdulaziz A. AlKhedhairy,^e^ Rais Ahmad Khan,^d,*^

***^a^****Department of Pharmacognosy, College of Pharmacy, King Saud University, P.O. Box 2457, Riyadh 11451, KSA.*

***^b^****Department of Chemistry, College of Science and General Studies, Alfaisal University, Riyadh, KSA.*

***^c^****Department of Food Science and Nutrition, Faculty of Food and Agricultural Sciences, King Saud University, 2460, Riyadh, 11451, KSA.*

***^d^****Department of Chemistry, College of Science, King Saud University, P.O. Box 2455, Riyadh 11451, KSA.*

*^e^Al-Jeraisy Chair for DNA Research, Zoology Department, College of Science, King Saud University, Riyadh 11451, KSA.*

**** Author for Correspondence:***

*Department of Chemistry, College of Science, King Saud University, P.O. Box 2455, Riyadh 11451, KSA. Mobile +966 536745404, Email:* [*raischem@gmail.com*](mailto:raischem@gmail.com) *(Khan, RA)*

**Experimental Section**

**Detection of apoptosis by flow cytometry**

Apoptosis was measured in MCF-7 cells by Annexin V- FITC and Propidium Iodide (PI) double staining method. Briefly, 1x10^5^ cells/ml were grown for overnight and exposed to complex **1** - **3** (50 µM), for 24 h in 6-well plates. At the end of the exposure, cells were washed with cold PBS, trypsinized and centrifuged at 1000 rpm; the cell pellet was rewashed with PBS and re-suspended in 100 µl of 1X binding buffer (1x10^6^ cells/ml). Then, Annexin V–FITC and PI, 5 µl each was added to the cell suspension, and the cells were gently vortexed. The cells were then incubated for 20 min at room temperature (25 °C) in the dark. Then, the samples were diluted by adding 400 µl 1X binding buffer. Annexin-V/ PI fluorescence was analyzed for each sample using a BD FACS Calibur flow cytometer. A total of 10,000 events were acquired for each sample and data were analyzed using Cell Quest Pro software (BD Biosciences).

**Figure S1:** FT-IR spectrum of the ligand, Schiff base.

**Figure S2:** FT-IR spectrum of the copper (II) complex **1**.

**Figure S3:** FT-IR spectrum of the copper (II) complex **2**.

**Figure S4:** FT-IR spectrum of the copper(II) complex **3**.

**Figure S5** EPR spectra of the complexes **2** and **3**


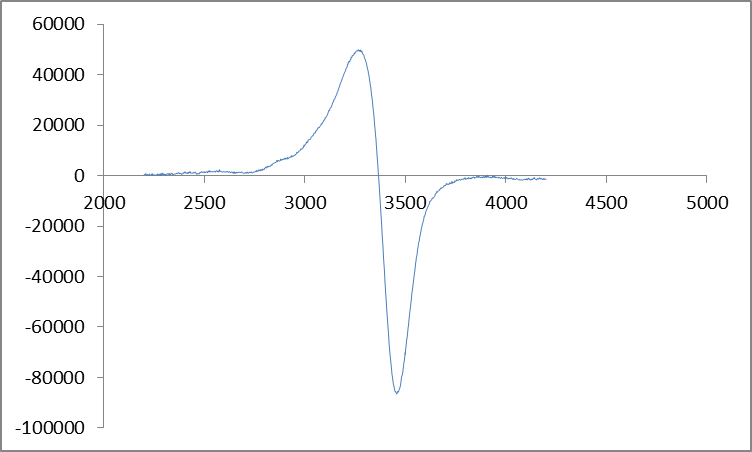

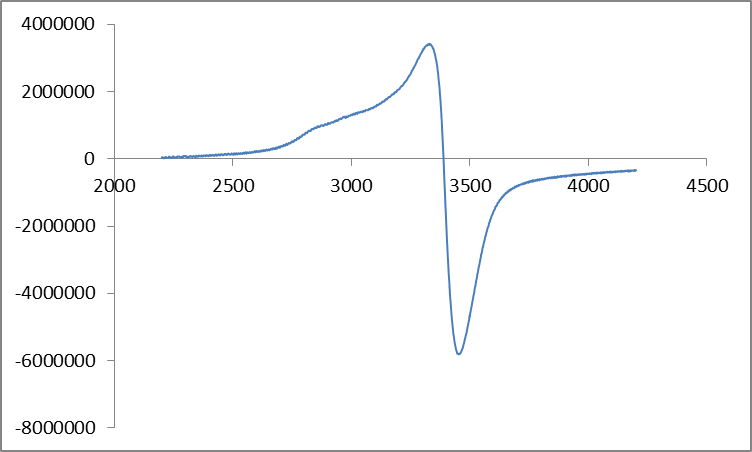


**Figure S6:** Quenching in the fluorescence of HSA in the presence of copper complexes **2** and **3**. *Inset* shows progressive decrease in the fluorescence intensity with increasing concentration of complexes **2** and **3**,


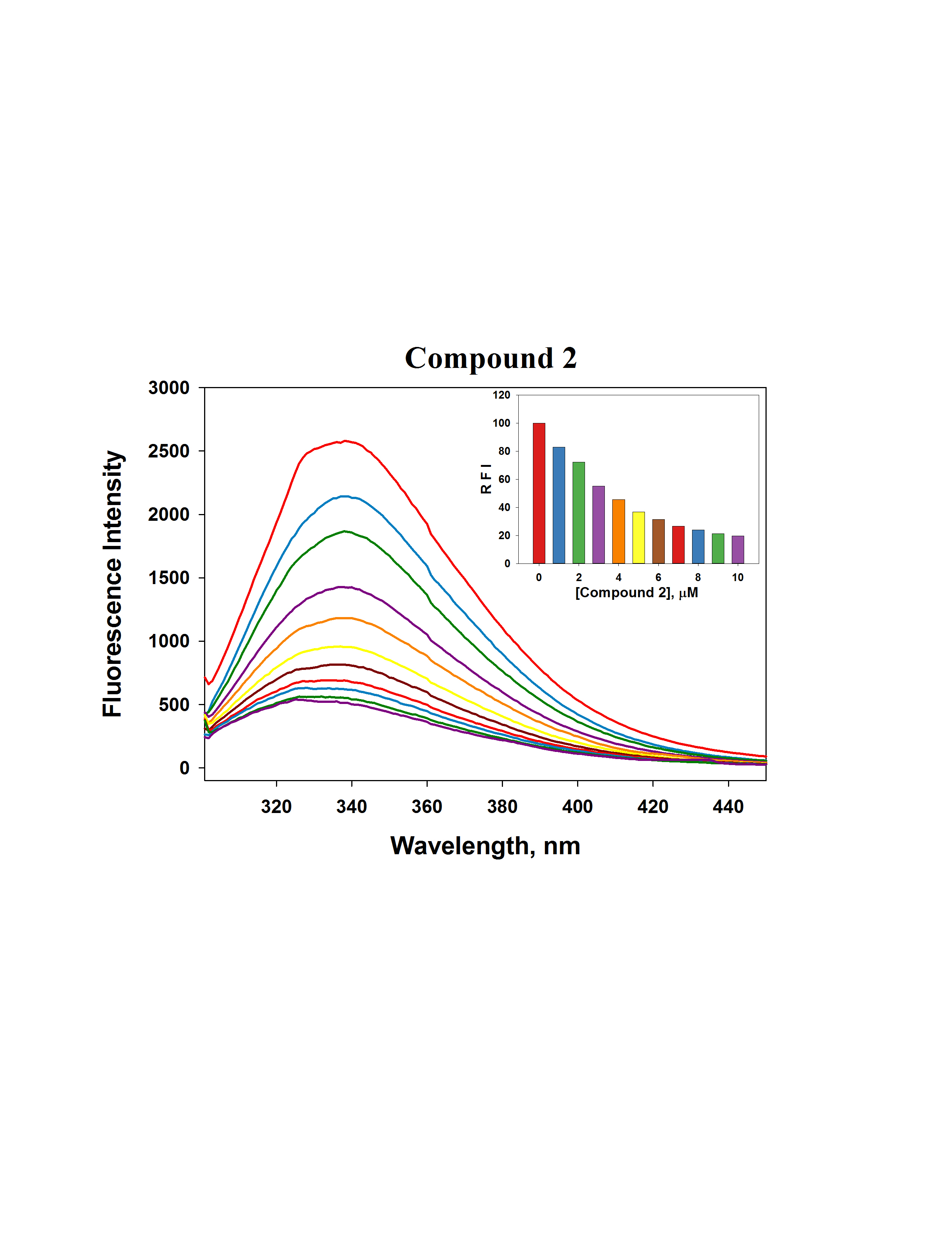

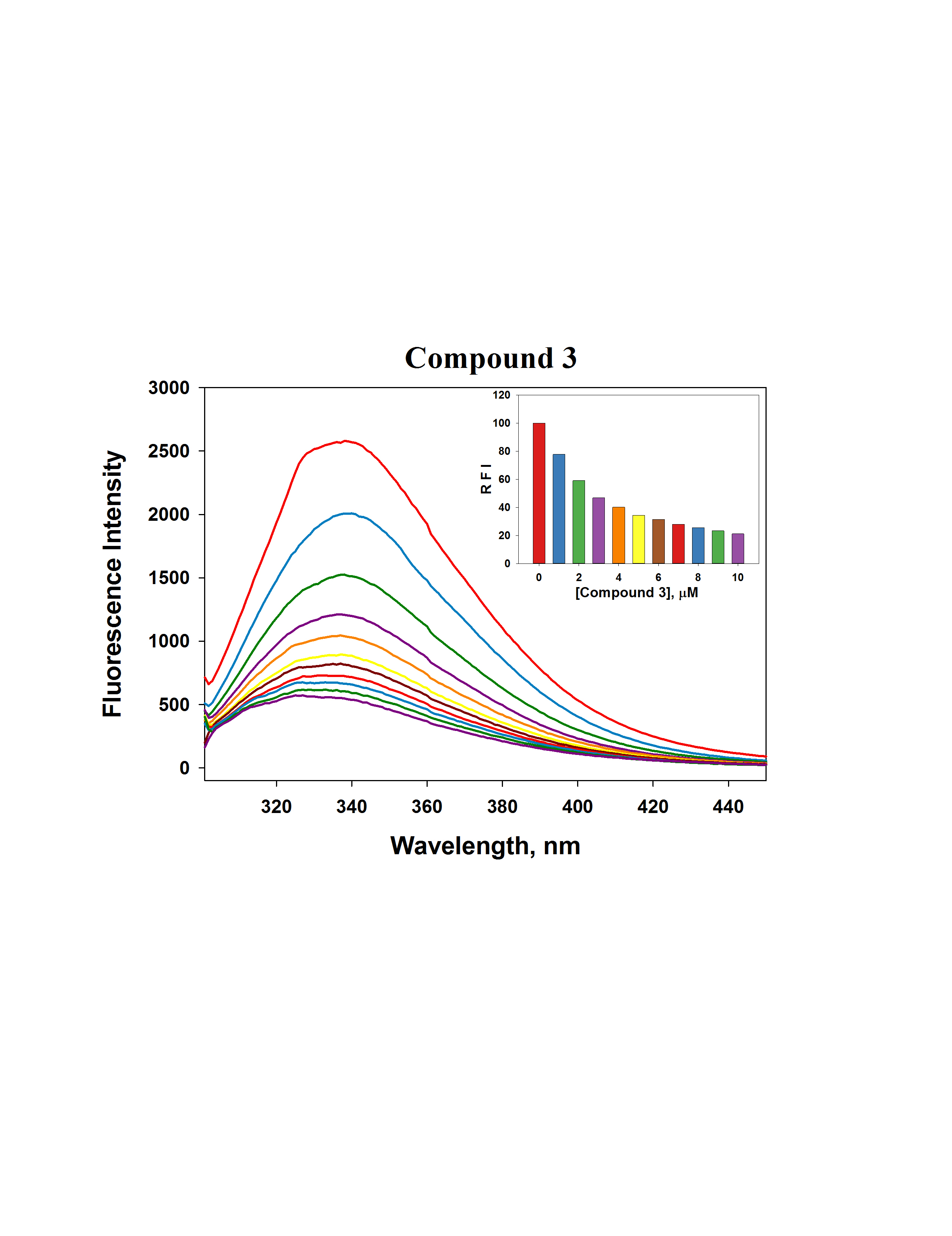


**Figure S7:** (a) Three-dimensional fluorescence of HSA in the presence and absence of metal complexes (b) Contour maps depicting three-dimensional fluorescence of HSA in the presence and absence of copper complexes **2** and **3**.


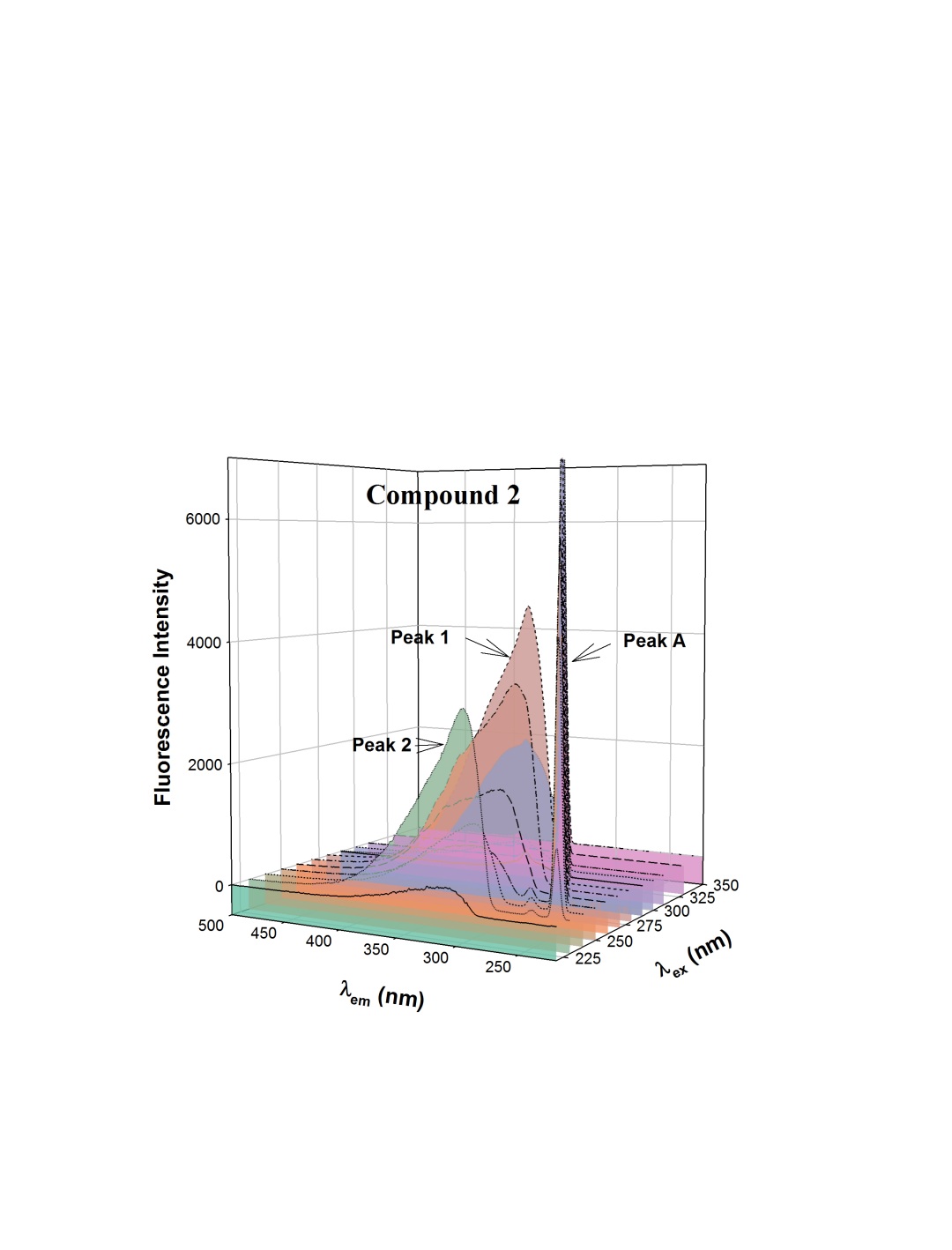

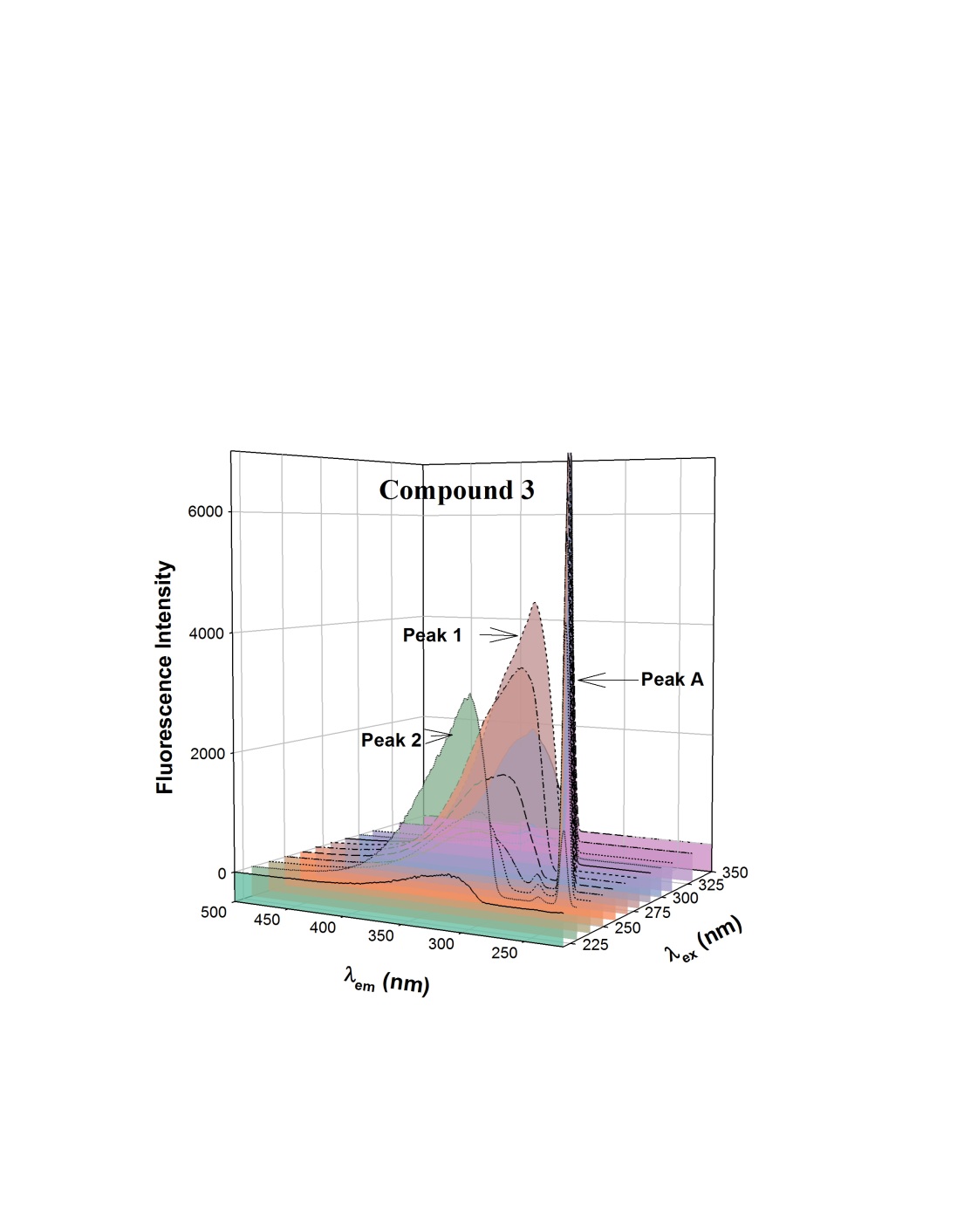

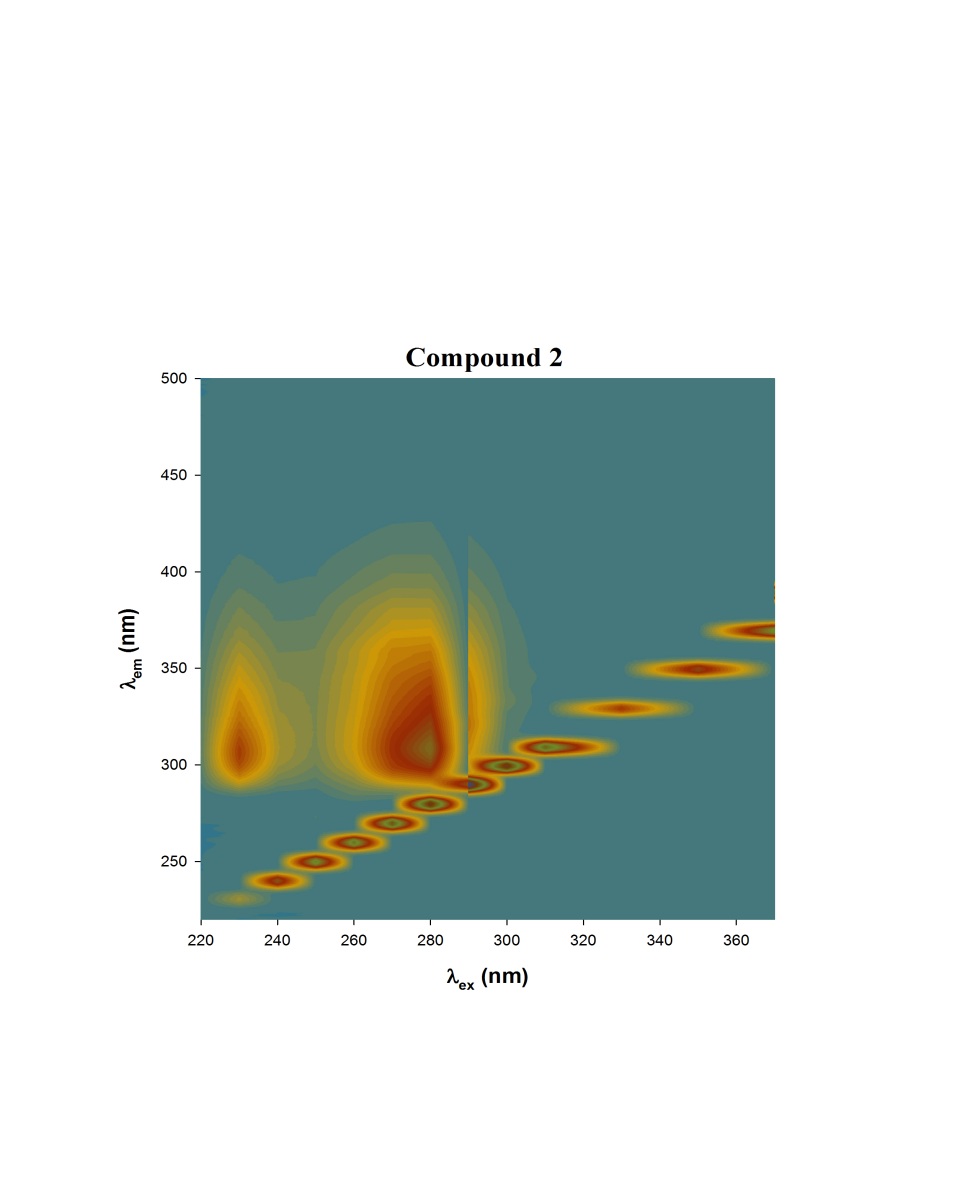

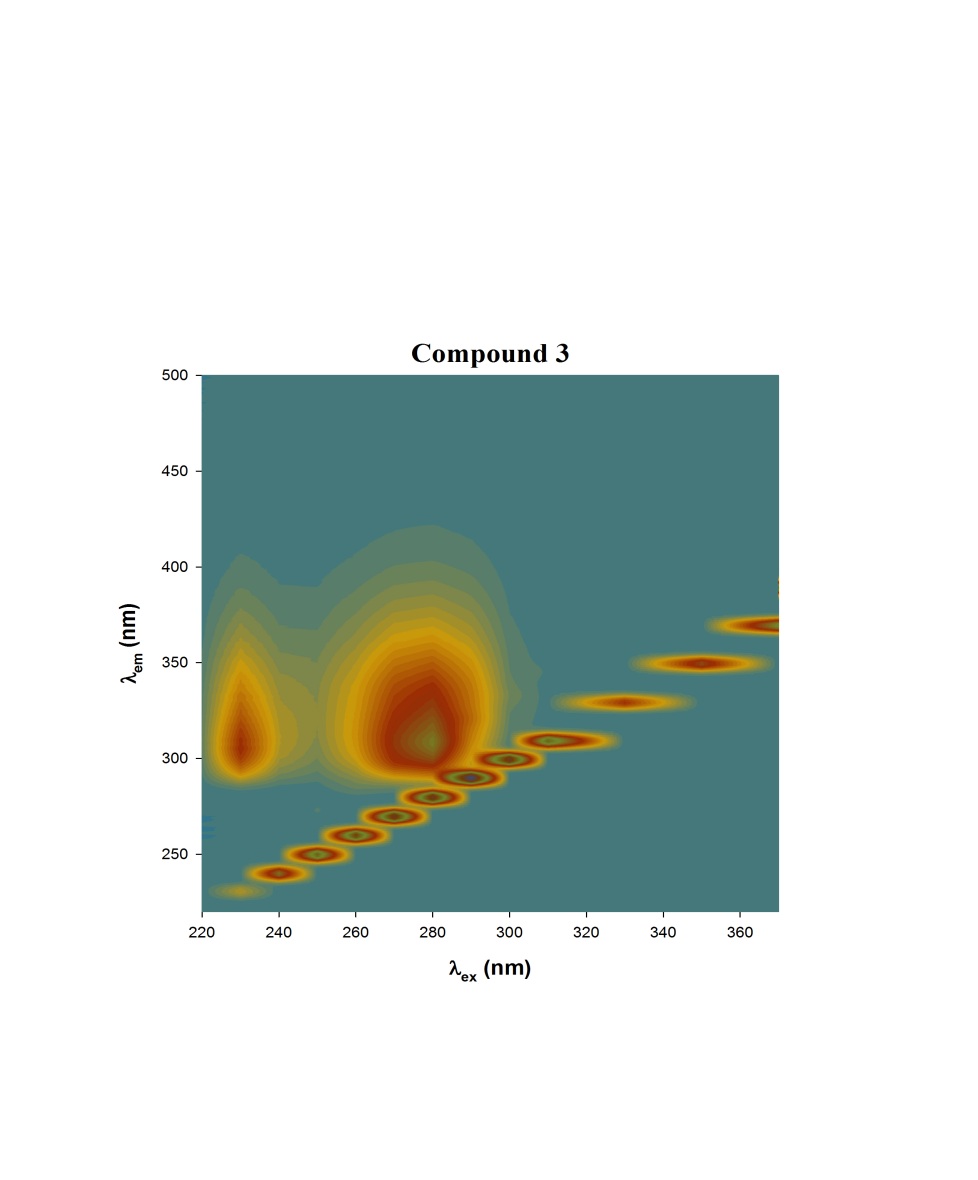


**Figure S8:** FRET between HSA and complexes **2** and **3**.


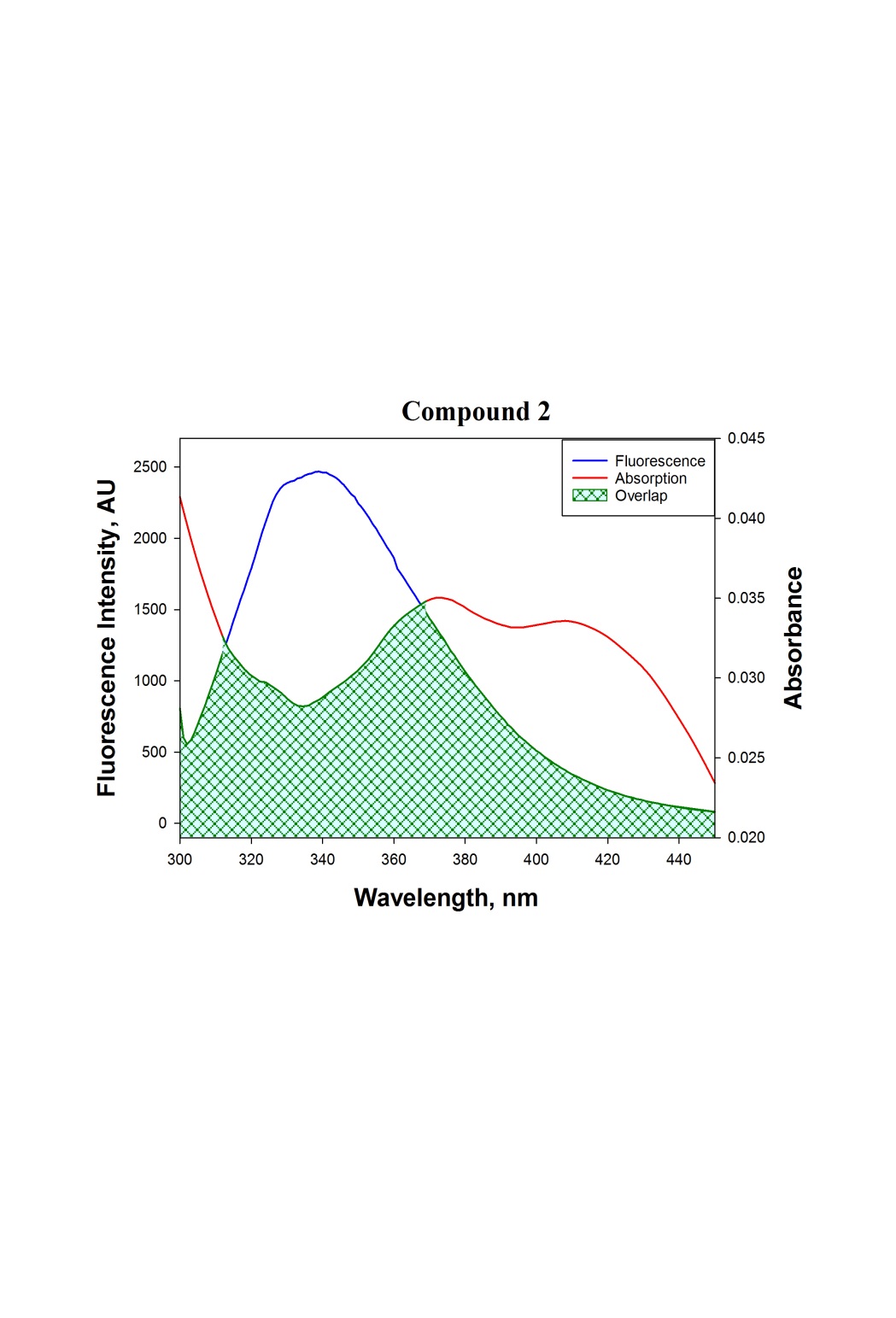

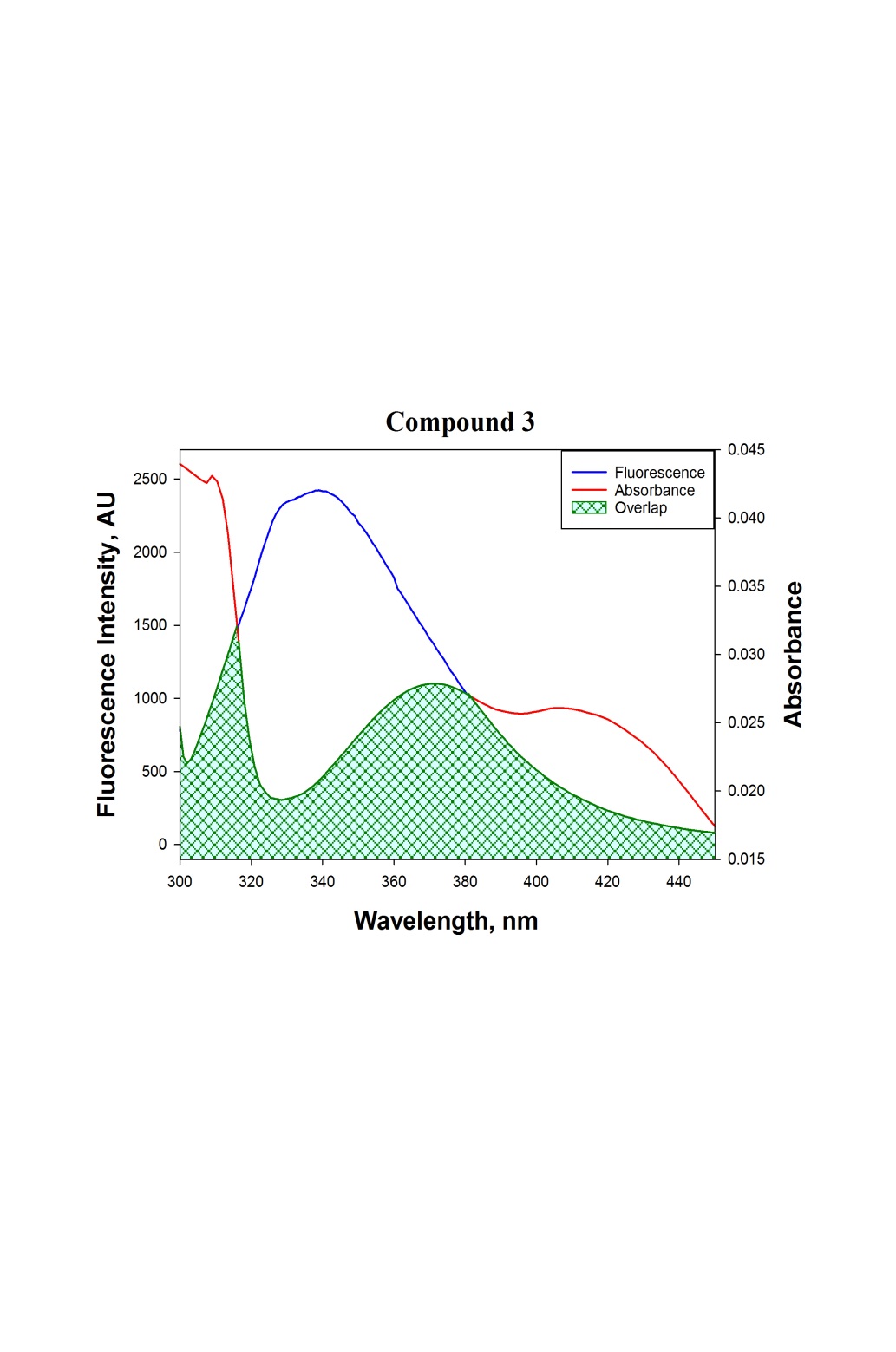


**Figure S9:** Cytotoxicity of the complexes 1-3 against HEK293 cells.


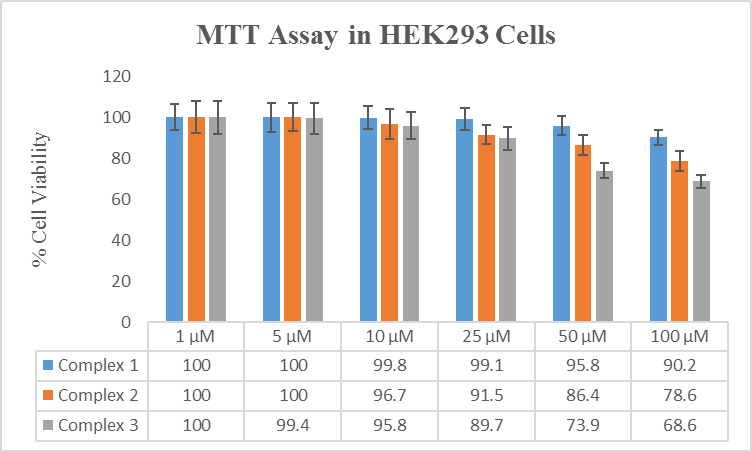


**Figure S10. (A)** Flow cytometric analysis (Annexin V-FITC/PI assay) of MCF-7 cells exposed to indicate concentration of complex **1**-**3** (50 μM) including an untreated control for 24 h. **(B)** Graph representing the apoptosis percentage observed. The data obtained from experiments were presented, as mean values, and the difference between control and test were analyzed using Student’s t-test. ∗p ≤ 0 05, ∗∗p ≤ 0 005, and ∗∗∗p ≤ 0 .001.


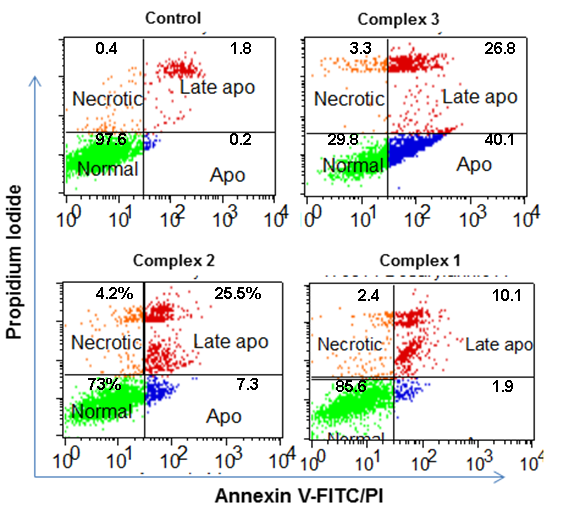


**Viable**

**Viable**

**Viable**

**Viable**

**(A)**

(B)

**Figure S11.** Fluorescence studies of ROS generation of complexes **1**-**3** using the DCFH-DA dye on MCF-7 cells. The data obtained from experiments were presented, as mean values, and the difference between control and test were analyzed using Student’s t-test.

∗p ≤ 0 05, ∗∗p ≤ 0 005, and ∗∗∗p ≤ 0 .001.
